# Nutritional stress compromises mosquito fitness and antiviral immunity, while enhancing dengue virus infection susceptibility

**DOI:** 10.1038/s42003-023-05516-4

**Published:** 2023-11-06

**Authors:** Jiayue Yan, Chang-Hyun Kim, Leta Chesser, Jose L. Ramirez, Chris M. Stone

**Affiliations:** 1 Illinois Natural History Survey, University of Illinois at Urbana–Champaign, Champaign, IL USA; 2 USDA, Agricultural Research Service, National Center for Agricultural Utilization Research, Crop Bioprotection Research Unit, Peoria, IL USA

**Keywords:** Entomology, Dengue virus

## Abstract

Diet-induced nutritional stress can influence pathogen transmission potential in mosquitoes by impacting life history traits, infection susceptibility, and immunity. To investigate these effects, we manipulate mosquito diets at larval and adult stages, creating two nutritional levels (low and normal), and expose adults to dengue virus (DENV). We observe that egg number is reduced by nutritional stress at both stages and viral exposure separately and jointly, while the likelihood of laying eggs is exclusively influenced by adult nutritional stress. Adult nutritional stress alone shortens survival, while any pairwise combination between both-stage stress and viral exposure have a synergistic effect. Additionally, adult nutritional stress increases susceptibility to DENV infection, while larval nutritional stress likely has a similar effect operating via smaller body size. Furthermore, adult nutritional stress negatively impacts viral titers in infected mosquitoes; however, some survive and show increased titers over time. The immune response to DENV infection is overall suppressed by larval and adult nutritional stress, with specific genes related to Toll, JAK-STAT, and Imd immune signaling pathways, and antimicrobial peptides being downregulated. Our findings underscore the importance of nutritional stress in shaping mosquito traits, infection outcomes, and immune responses, all of which impact the vectorial capacity for DENV transmission.

## Abstract (web summary)

An experimental study on the yellow fever mosquito reveals that inadequate nutrition during both the larval and adult stages could mediate their vectorial capacity by compromising key life history traits and antiviral immune response, while boosting vulnerability to dengue infection.

## Introduction

The fitness and performance of organisms have been a significant focus of biological research, often assessed through their phenotypic traits^1^. Phenotypic traits such as body size, survival, and fecundity, while exhibiting low heritability, demonstrate high plasticity, rendering them susceptible to environmental changes^2,3^. Notably, environmental factors like food sources, which are often limited but greatly variable across diverse habitats, can play a pivotal role in shaping phenotypic traits. Changes in food quantity or quality can induce nutritional stress, impacting traits, a phenomenon especially pronounced in insects undergoing metamorphosis, where body structures undergo remodeling in response to abiotic and biotic influences^4–6^. Insects such as mosquitoes, with a larval aquatic stage reliant on microorganisms and organic detritus as nutrition and an adult terrestrial stage relying on plant sugars and vertebrate blood^7,8^, offer an excellent model to investigate these effects. Larval environmental conditions, including temperature, competition, and microbiota composition, can shape adult phenotypes through “carry-over effects^9–11^”. Concurrently, conditions in adult habitats such as air temperature, humidity, plant, and microbial diversity directly influence phenotypic traits^12–15^. The disparities between larval and adult environments, coupled with selective pressures, can engender diverse phenotypic responses to identical factors (e.g., immunity against bacterial infection^16^). Despite its significance, the role of nutritional stress from larval and adult diets in shaping mosquito phenotypic traits remains understudied. Specifically, there is a scarcity of research that combines the effects of cross-stage nutritional stress on adult phenotypes.

The vectorial capacity of mosquito vectors for human pathogens hinges directly on their adult life history traits. Vectorial capacity is a measure of a mosquito population’s potential for transmitting vector-borne pathogens and is based on several entomological parameters including survival and—indirectly, through its effect on population size—fecundity^17^. Nutritional stress mediated by larval and adult diets can indirectly influence vectorial capacity by affecting phenotypic traits. For instance, the body size of female *Aedes aegypti* has been demonstrated to be negatively associated with their susceptibility to dengue virus (hereafter ‘DENV’) infection and dissemination^18^. Adult survival is vital for vectorial capacity since mosquitoes must survive long enough to enable substantial pathogen replication, body-wide dissemination, and salivary gland entry prior to transmission in subsequent bites (i.e., surviving the extrinsic incubation period)^19^. Fecundity and survival collectively shape the mosquito’s lifetime reproductive output, consequently influencing local mosquito density—a parameter within the vectorial capacity equation.

Beyond life history traits, the transmission of vector-borne pathogens is contingent on vector competence, reflecting the probability of a mosquito getting infected after ingesting an infectious blood meal and subsequently transmitting the pathogen in subsequent bites^20^. The mosquito immune response, a pivotal determinant of vector competence^21^, operates through hemocytes, barrier defenses, constitutive defenses, and four canonical immune signaling pathways—of which three combat arbovirus infections in mosquitoes^22,23^. Among these, the functions of the NF-κB Toll and the immune deficiency (Imd), and the Janus kinase/signal transducers and activators of transcription (JAK-STAT) pathways are well documented^24–28^. Elicitation of the Toll and Imd immune signaling pathways results in the synthesis and secretion of antimicrobial effector molecules, including antimicrobial peptides (AMPs), which directly combat invading microbial pathogens^24^. Despite its critical role, the influence of different nutritional stresses on immune signaling pathways remains largely unexplored^29^.

Given the pivotal role of nutrition in fecundity and longevity, nutrition-induced stress potentially influences vectorial capacity. For instance, larval food quantity has been demonstrated to impact *Anopheles* mosquitoes’ vectorial capacity for transmitting the human malaria parasite^30^, while nectar availability was shown to affect the vectorial capacity of *An. gambiae*^31^. In addition, nutrition might modulate vectorial capacity by affecting susceptibility to pathogen infections and mosquito immunity. Although previous studies have explored the effect of sugar feeding on arboviral infections, the effects of both larval and adult nutritional stress on DENV infection susceptibility remain unclear^32,33^. Earlier studies by Telang et al.^29^ and Caragata et al.^34^ have investigated the role of larval and adult diets in the expression of key immune genes, suggesting either a positive effect of larval nutritional stress or a negligible effect of adult diet on immune gene expression. However, the combined influence of cross-stage nutritional stress on immunity, and whether the effects of stress during these two life stages are intertwined or separate, remain relatively unexplored.

**Fig. 1:**
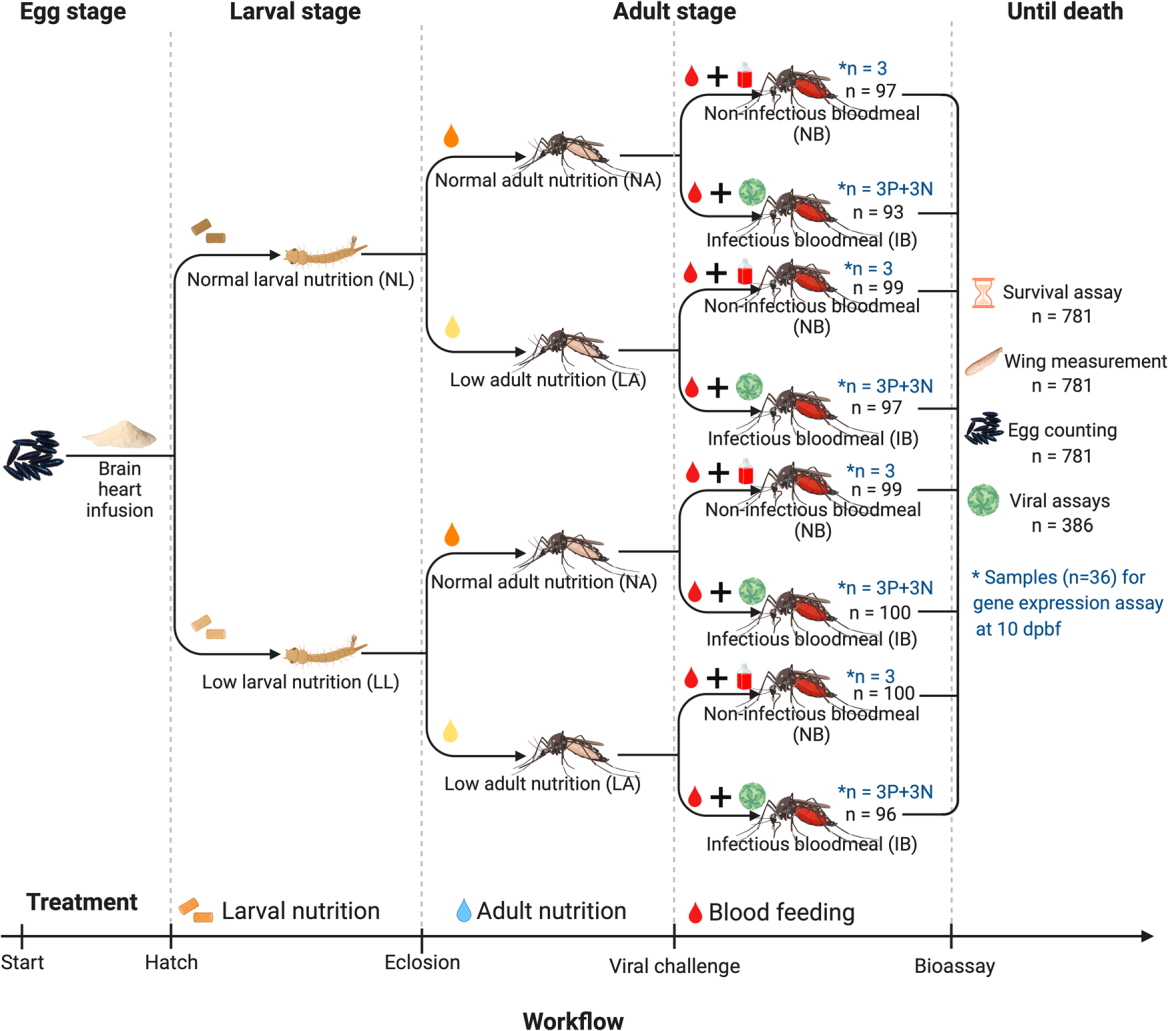
Overview of experimental design. Brain heart infusion is the powder used for egg hatching. Normal or low larval nutrition represents an access to larval food (100 mg of rabbit chow: lactalbumin: yeast at 1:1:1 ratio) on days 2, 4, 5, and 6 post hatch or on days 2 and 6 post hatch. Normal or low adult nutrition represents an ad libitum access to 10% or 1% sucrose solution daily. Infectious or noninfectious blood meal represents a one-time access to a mixture of bovine blood and cell culture medium containing dengue-4 virus (1:1) or a mixture of bovine blood and cell culture medium (1:1). The treatments were performed in two subsequent blocks and n (in black) represents the combined sample size for each treatment level. Additional 36 engorged mosquitoes that have gone through the same treatments were processed to gene expression assay at 10 days post blood-feeding: *n (in blue) represents the sample size for gene expression assay, while P or N represents positive or negative dengue-4 virus infection. Each group of noninfectious blood meal was used as the reference for the infectious blood meal group in the same brackets in the calculation of relative fold change of gene expression. Created with BioRender.com.

This study investigates the effects of nutritional stress during larval and adult stages, and their interaction, on phenotypic traits including life history traits, susceptibility to DENV infection, and immune gene expression in the mosquito *Ae. aegypti*. This mosquito species serves as the primary vector for arboviruses of public health significance, including dengue, yellow fever, Zika, and Chikungunya viruses. As nutrition is a requirement for mosquito growth, reproduction, and performance, we hypothesize that nutritional stress will negatively affect adult traits, infection susceptibility, and immunity. To test our hypothesis, we manipulated larval food amount and adult sugar concentration to establish two nutritional levels (normal vs. low) for each stage, followed by exposing adults to either a dengue-infectious blood meal or a noninfectious blood meal (Fig. 1). We then measured and compared adult size, survival and fecundity, dengue viral status and titer, and immune gene expression against DENV infection in *Ae. aegypti* between the two nutritional levels during larval and adult stages.

## Results

### Life history traits

We first investigated how nutritional stress affected *Ae. aegypti* life history traits. The mean wing length, fecundity, and survival of mosquitoes reared at two levels of larval and adult nutritional treatments (referred to as “NL”, “LL”, “NA”, “LA”, indicating normal/low larval/adult nutrition) and exposed to either a noninfectious blood meal or dengue-infectious blood meal (“NB” or “IB”) are presented in Table 1. Mosquitoes reared at LL exhibited notably smaller sizes compared to those under NL, with mean wing sizes of 2.58 mm ± 0.01 and 2.83 mm ± 0.01, respectively (*t* = −26.87, *P* < 0.001). As expected, adult nutrition had no effect on mosquito size independently (*t* = 1.24, *P* = 0.22) or interactively with larval nutrition (*t* = 1.32, *P* = 0.19).

**Table 1:** Life history traits of *Aedes aegypti*.

Larval nutrition	Adult nutrition	Blood-feeding	Wing length	Fecundity	Survival
NL	NA	NB	2.82 ± 0.01 SE	70.58 ± 2.01 SE	30.19 ± 1.52 SE
		IB	2.81 ± 0.01 SE	67.43 ± 2.03 SE	33.45 ± 1.58 SE
	LA	NB	2.82 ± 0.01 SE	45.22 ± 3.02 SE	25.32 ± 1.42 SE
		IB	2.83 ± 0.01 SE	32.88 ± 3.15 SE	28.72 ± 1.24 SE
LL	NA	NB	2.57 ± 0.01 SE	43.38 ± 1.65 SE	31.73 ± 1.65 SE
		IB	2.56 ± 0.01 SE	39.94 ± 2.01 SE	30.36 ± 1.38 SE
	LA	NB	2.59 ± 0.01 SE	21.67 ± 2.60 SE	18.20 ± 1.33 SE
		IB	2.59 ± 0.01 SE	13.94 ± 2.24 SE	20.24 ± 1.58 SE

*NL* normal larval nutrition, *LL* low larval nutrition, *NA* normal adult nutrition, *LA* low adult nutrition, *SE* standard error.

Mean wing length is recorded to 2 decimal places in mm and measured as described in the main text. Mean fecundity is represented by the number of eggs laid. Mean survival is the number of days that the individual lived post blood-feeding.

**Fig. 2:**
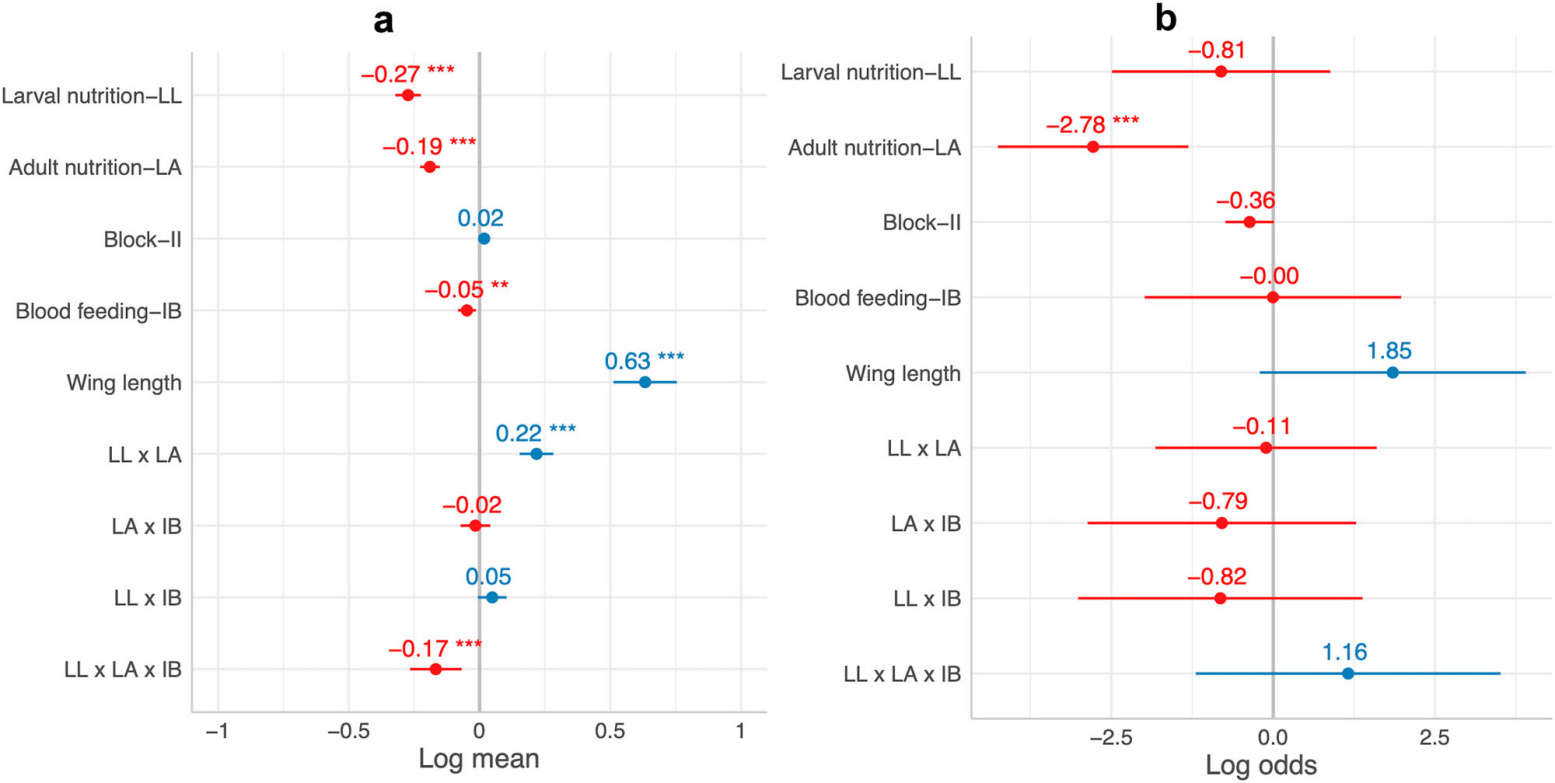
Hurdle model explaining the variations in fecundity of *Aedes aegypti*. In the Poisson model for egg count (**a**), low larval nutrition (LL), low adult nutrition (LA), and infectious blood meal (IB) had a negative effect on fecundity, while wing length positively affected fecundity. The interaction between LL and LA and the three-way interaction between, LL, LA, and IB also significantly affected fecundity, with the combined effect greater than the sum of their single effects in the former but the opposite for the latter. In the binomial model for egg presence/absence (**b**), only LA had a significantly negative effect. Numbers and dots in the plots represent estimates and the length of each line passing through the dots represents standard error. *** and ** represent significant level at 0.001 and 0.01. The gray lines represent neutral effects.

We utilized hurdle models to analyze variations in fecundity, considering both egg presence/absence and egg count data. Mosquitoes experiencing nutritional deprivation during larval (29.93 ± 1.24 SE vs. 53.98 ± 1.53 SE) or adult stages (28.5 ± 1.51 SE vs. 55.31 ± 1.18 SE), or subjected to an IB challenge (38.32 ± 1.54 SE vs. 45.25 ± 1.47 SE), laid significantly fewer eggs than their counterparts. Larger mosquitoes tended to lay more eggs (Fig. 2a). Notably, the positive LL and LA interaction (Fig. 2a) indicated a more pronounced reduction in egg count due to joint larval and adult nutritional deprivation compared to separate deprivation (i.e., synergistic effect). While the effect of infectious blood meal (Fig. 2a) was significant, it was relatively weak, implying that viral challenge alone did not induce substantial reproductive costs here. When considering this effect alongside LL and LA in a three-way interaction, a significant negative impact emerged (Fig. 2a), suggesting that the reduction in fecundity due to joint larval and adult nutritional deprivation could be partly offset by blood meal intake, especially when the infectious blood meal alone has limited fitness costs. Egg presence or absence (Fig. 2b) was strongly and negatively affected solely by LA, suggesting a potential threshold of adult energy levels triggering egg-laying.

**Fig. 3:**
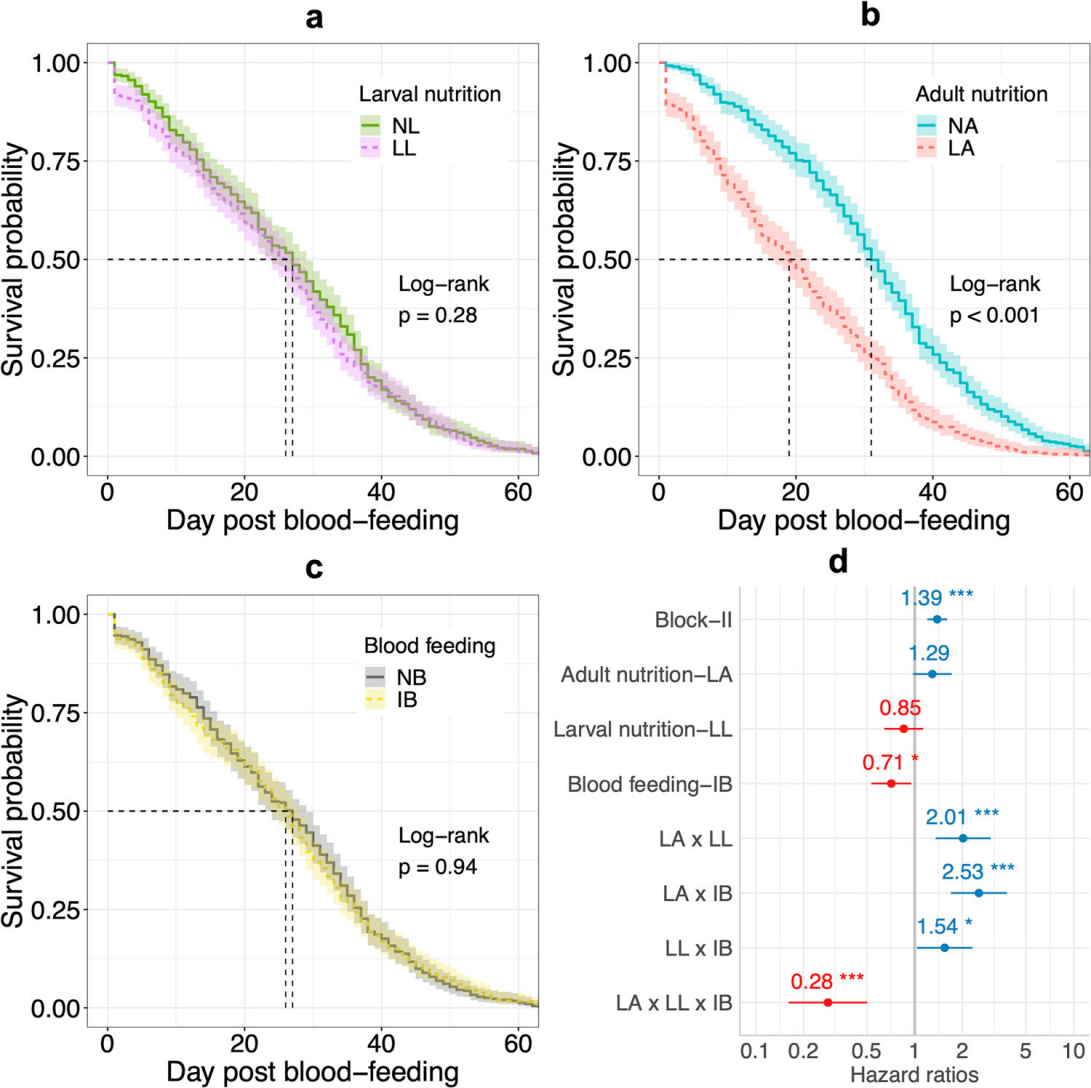
Survival models explaining the variations in survival of *Aedes aegypti*. In the survival curves for different levels of larval nutrition (**a**), adult nutrition (**b**), and blood-feeding (**c**), while there was no significant difference between normal (NL) and  low (LL) larval nutrition nor between infectious (IB) and noninfectious (NB) blood meal, low adult nutrition (LA) showed a negative impact on survival probabilities compared to normal adult nutrition (NA). In the Cox Proportional Hazards model (**d**), IB had a negative impact on survival; the interaction between LA and LL, between LA and IB, and between LL and IB had a greater combined effect than the sum of their single effects, while the opposite was true for the three-way interaction between LA, LL and IB. Numbers and dots in plot A represent estimates and the length of each line passing through the dots represents standard error. *** and ** represent significant level at 0.001 and 0.01. The gray line represents neutral effects. Survival probabilities were estimated by the Kaplan–Meier method and shadow areas represent 95% confidence intervals. The dotted line represents day at median survival for each treatment level (NL = 27, LL = 26, NA = 31, and LA = 19, NB = 27, IB = 26).

To assess the effects of larval and adult nutritional levels and blood-feeding on survival, separate survival curves were fitted for these three treatment terms. while low larval nutrition had no significant effect (Fig. 3a), mosquitoes reared at low concentration of sucrose solution (1%) exhibited lower mean survival compared to those with normal sucrose concentration (10%) (Fig. 3b). Importantly, the ingestion of an infectious blood meal did not significantly diminish mosquito survival (Fig. 3c), indicating that viral exposure alone had minimal impact on longevity. Further examining their interactions using Cox Proportional Hazards (CPH) modeling, neither LL nor LA alone significantly affected survival (Fig. 3d). However, the inclusion of nutritional stress and their interactions made the effect of an infectious blood meal (IB) significant, indicating that viral challenge’s fitness cost on survival could be intensified in combination with other factors. All interactions significantly affected survival (Fig. 3d), notably the positive interactions of LL and LA, LL and IB, as well as LA and IB, indicating that combined larval and adult nutritional deprivation, along with either deprivation paired with viral challenge, synergistically reduced mosquito survival. Consistent with their effects on fecundity, the three-way interaction of LL, LA, and IB had a relatively weaker impact on survival compared to two-way interactions, suggesting that a blood meal could partially compensate for severe fitness costs stemming from joint larval and adult nutritional deprivation. Neither Mosquito size nor its interaction with larval nutrition affected survival. Collectively, adult nutritional stress could reduce mosquito longevity, yet larval nutrition and an infectious blood meal could mask its independent effect due to more substantial interactive effects. Furthermore, the ingestion of a dengue-infectious blood meal, in conjunction with nutritional deprivations, could jointly reduce mosquito longevity.

**Fig. 4:**
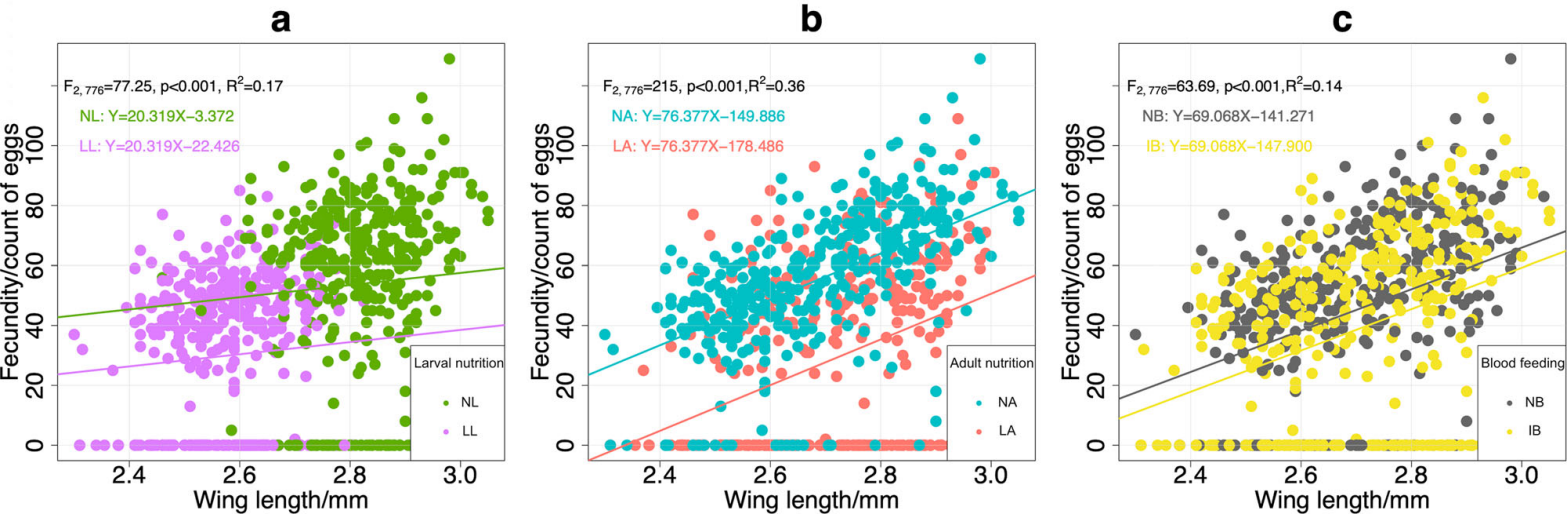
Regression relationship between fecundity and wing length. The regression was performed to compare mosquitoes from two levels of larval nutrition (**a**), adult nutrition (**b**), and blood-feeding (**c**), respectively. Analysis of covariance (ANCOVA) showed that the positive relationship between fecundity and wing length did not change at different levels of larval nutrition (slope = 20.32), adult nutrition (slope = 76.38) or blood-feeding (slope = 69.07). The effects of treatments on fecundity after controlling for the effect of body size (wing length) were significant (larval nutrition: *F*_2__,__776_ = 77.25, *P* < 0.001, *R*^2^ = 0.17; adult nutrition: F_2__,__776_ = 215, *P* < 0.001, *R*^2^ = 0.36; blood-feeding: *F*_2__,__776_ = 63.69, *P* < 0.001, *R*^2^ = 0.14). NL normal larval nutrition, LL low larval nutrition, NA normal adult nutrition, LA low adult nutrition.

There was a positive correlation between mosquito fecundity and size (estimate ± SE = 69.37 ± 6.48, *t* = 10.71, *P* < 0.001, *R*^2^ = 0.13). We performed the analysis of covariance (ANCOVA) to further explore whether this relationship differs across treatment levels. We found it remained consistent across the three treatments, while each treatment significantly influenced fecundity even after accounting for mosquito size (Fig. 4a–c).

### DENV infection and dissemination

We proceeded to evaluate whether nutritional stress could alter the susceptibility of mosquitoes to DENV infection and dissemination, comparing those reared at LL or LA with counterparts reared at NL or NA. Mosquito bodies and legs were dissected and tested for dengue viral infection prevalence and titer using RT-qPCR. Infection in bodies and dissemination to legs were used as proxies for infection and dissemination, respectively. Dengue viral prevalence and mean viral titer (log 10 transformed) in bodies and legs of *Ae. aegypti* are presented in Table 2. Mosquitoes reared at LL and LA exhibited higher infection and dissemination rates than their counterparts, while infected individuals from LL and disseminated individuals from both LL and LA displayed lower mean viral titer than their counterparts. We extended our analysis by utilizing a Generalized Linear Model (hereafter “GLM”), which incorporated mosquito size and survival along with their interactions, to examine the influence of nutritional stress on dengue viral prevalence and titer.

**Table 2:** Dengue viral prevalence and titer in *Aedes aegypti*.

Larval nutrition	Adult nutrition	Blood-feeding	Prevalence B	Prevalence L	Titer B	Titer L
NL	NA	NB	0	0	0	0
		IB	9.68 %	7.53 %	6.50 ± 0.17 SE	5.07 ± 0.42 SE
	LA	NB	0	0	0	0
		IB	48.98 %	34.69 %	6.10 ± 0.21 SE	4.36 ± 0.30 SE
LL	NA	NB	0	0	0	0
		IB	24.00 %	15.00 %	5.72 ± 0.39 SE	5.22 ± 0.31 SE
	LA	NB	0	0	0	0
		IB	61.46 %	40.63 %	5.78 ± 0.19 SE	4.51 ± 0.26 SE

*NL* normal larval nutrition, *LL* low larval nutrition, *NA* normal adult nutrition, *LA* low adult nutrition, *B* body, *L* leg, *SE* standard error.

Viral prevalence is calculated as the number of positive samples at a treatment level divided by the total number of samples that had fed with infectious blood meal at that level. Viral titer is measured using all the positive samples at each level and presented as a log 10 value in the Table. In all, 12 outliers exceeded 1.5 IQR were removed from viral titer in mosquito body.

**Fig. 5:**
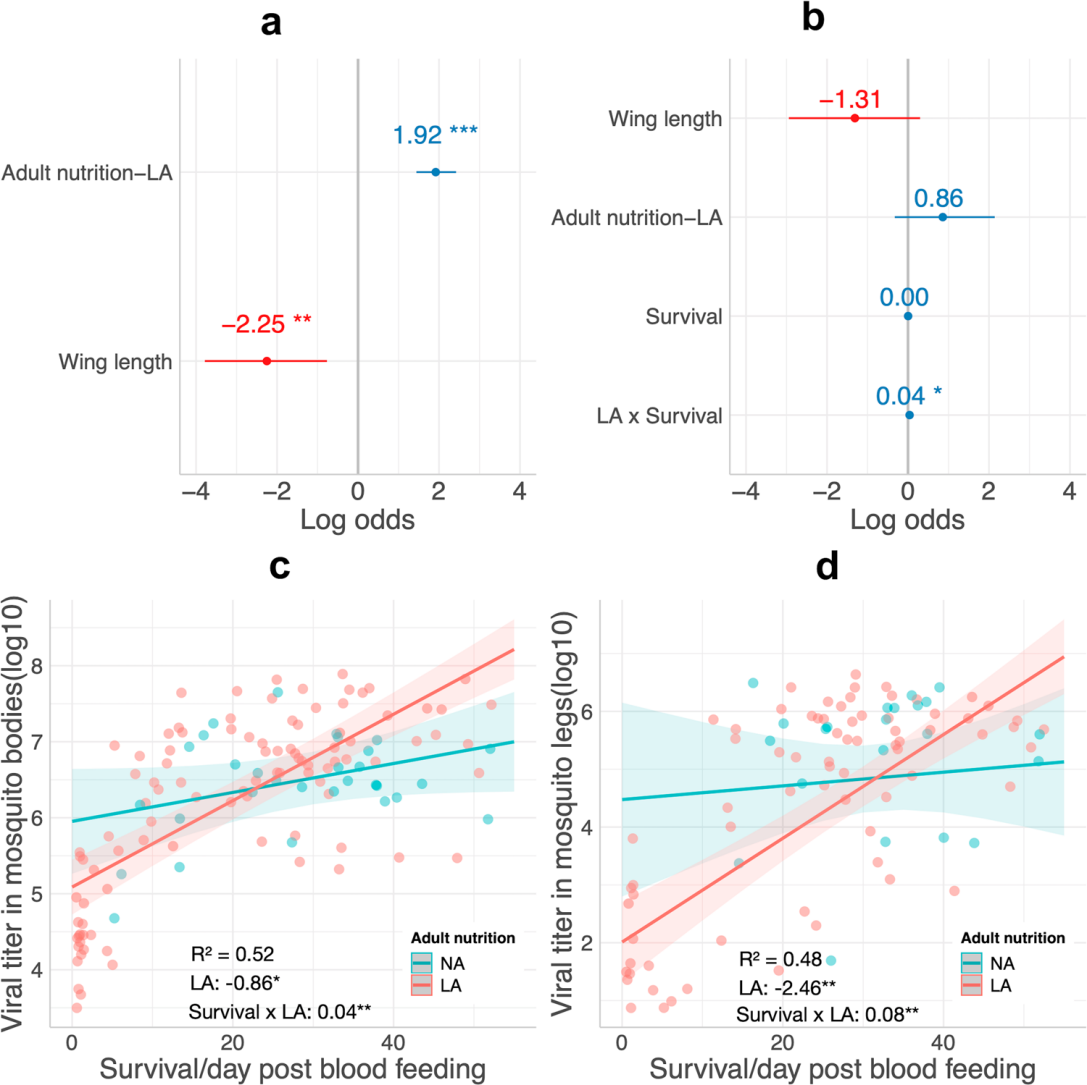
Models explaining the variations in dengue viral status (prevalence) and titer (intensity) of *Aedes aegypti*. In the model for infection status (**a**), low adult nutrition (LA) had a positive effect, while wing length showed a negative effect. In the model for dissemination status (**b**), only the interaction between LA and survival length had a significant effect, and their combined effect was larger than the sum of their single effects. In the model for viral titers in mosquito body (**c**), LA had a negative effect and the combined effect of survival and LA interaction was slightly stronger than their single effects. In the model for viral titers in mosquito legs (**d**), similar effects as last model were found. Numbers and dots in the plot A and B represent estimates and the length of each line passing through the dots represents standard error. ***, **, and * represents significant level at 0.001, 0.01, and 0.05, respectively. The gray lines represent neutral effects. NA normal adult nutrition, LA low adult nutrition.

Our findings revealed that adult nutritional stress increased mosquito infection susceptibility. Interestingly, while larval nutrition stress did not exert a significant effect, smaller mosquitoes demonstrated significantly higher infection rates (Fig. 5a; *R*^2^ = 0.14). This suggests that larval nutrition indirectly influences infection probability through its impact on mosquito body size. The impact of nutritional stress on dissemination rates was comparatively milder. A significant effect emerged from the interaction between adult nutrition and time post blood-feeding on dissemination rate, with no significant main effects (Fig. 5b; *R*^2^ = 0.13). Dengue viral titers in both bodies (*R*^2^ = 0.52; Fig. 5c) and legs (*R*^2^ = 0.48; Fig. 5d) yielded parallel outcomes. Adult nutritional stress displayed a significant negative impact, while the combined effect of the interaction between adult nutritional stress and survival was slightly more pronounced than their individual effects. This suggests that, even under nutritional stress, mosquitoes that managed to survive longer could develop higher titers. Consequently, adult nutritional stress assumes a pivotal role in determining dengue viral infection and dissemination prevalence and titer, with its effect covarying with mosquito size and longevity.

### Immune gene expression

We explored whether nutritional stress influences a mosquito’s immune response against DENV infection. To achieve this, we subjected *Ae. aegypti* to the same nutritional treatments as in previous experiments and exposed them to a dengue-infectious blood meal. We compared this to a reference group that received a noninfectious blood meal, allowing us to calculate relative fold change in the expression of 14 immune genes in both positive and challenged but negative samples. Utilizing GLMs, we assessed how larval nutrition, adult nutrition, DENV infection status (positive or negative), and their interactions impact the expression of each immune gene.

**Fig. 6:**
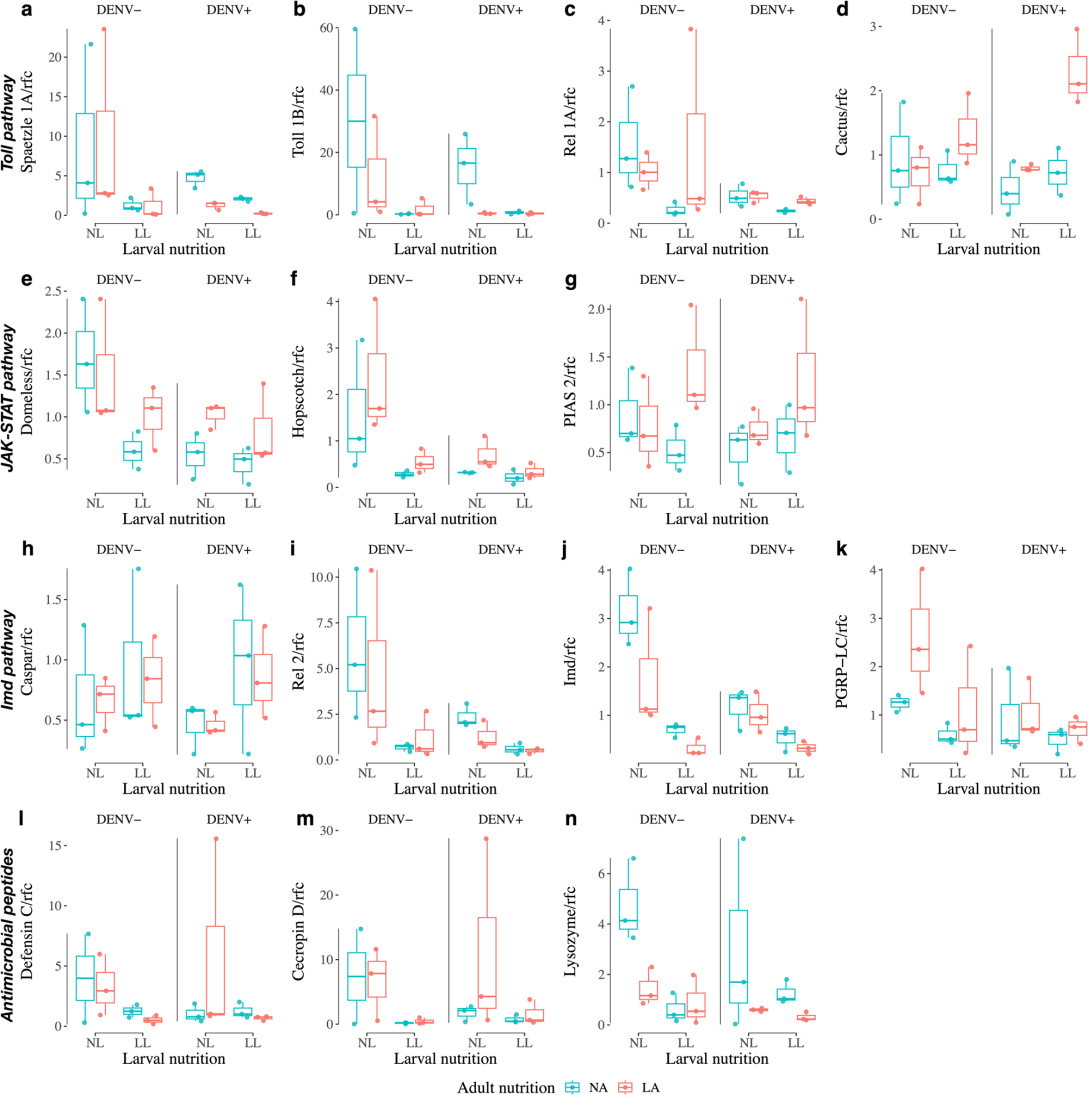
Immune gene expression of *Aedes aegypti* under nutritional stress and post DENV challenge. In Toll pathway, the expression of Spaetzle 1 A (**a**) and Toll 1B (**b**) were negatively affected by low larval nutrition (LL), while no effect for Rel 1 A (**c**) and Cactus (**d**). In JAK-STAT pathway, the expression of Domeless (**e**) and Hopscotch (**f**) were negatively affected by both LL and positive dengue virus infection (DENV +), while no effect for PIAS 2 (**g**). In Imd pathway, the expression of Rel 2 (**i**), Imd (**j**), and PGRP-LC (**k**) were negatively affected by LL; the expression of both Rel 2 (**i**) and Imd (**j**) were also negatively affected by DENV+; low adult nutrition (LA) also negatively affected the expression of Imd (**j**); no effect for the expression of Caspar (**h**). In antimicrobial peptides, the expression of Cecropin D (**m**) and Lysozyme (**n**) were negatively affected by LL; LA also negatively affected the expression of Lysozyme (**n**); no effect for the expression of Defensin C (**l**). Relative gene expression was measured using the delta–delta Ct method. NL normal larval nutrition, NA normal adult nutrition, DENV- negative dengue virus infection. The line within each box indicates the median and the edges of each box the first (Q1) and third (Q3) quartiles; the whiskers extend over 1.5 times the interquartile range.

Our gene expression analysis involving components and effector genes from three immune pathways revealed the downregulation of 9 out of 14 genes due to larval nutritional stress. Notably, the Toll pathway displayed suppressed activity as evidenced by the significant downregulation of upstream components Spaetzle (Fig. 6a and Table 3) and Toll (Fig. 6b and Table 3). Similar effects were observed in the Imd pathway, with significant downregulation of Imd (Fig. 6j and Table 3), PGRP-LC (Fig. 6k and Table 3), and the transcription factor Rel 2 (Fig. 6i and Table 3) in mosquitoes subjected to larval nutritional stress. Corroborating these results, larval nutritional stress also led to reduced expression of Cecropin (Fig. 6m and Table 3) and Lysozyme (Fig. 6n and Table 3), both crucial antimicrobial peptides regulated by of these immune pathways. Moreover, the JAK-STAT pathway demonstrated diminished activity as indicated by the downregulation of Domeless (Fig. 6e and Table 3) and Hopscotch (Fig. 6f and Table 3), upstream components, in larval nutritionally stressed mosquitoes.

**Table 3:** Generalized linear models explaining the variations in immune gene expression of *Aedes aegypti*.

Responses	Toll pathway				JAK-STAT pathway			Imd pathway				Antimicrobial peptides		
Predictors	Spa	Tol	Rel 1	Cac	Dom	Hop	PIA	Cas	Rel 2	Imd	PGRP	Def	Cec	Lyso
LL	*	*	–	NS	**	**	–	NS	**	***	*	NS	*	**
	–4.86	–12.3	–	0.05	–0.80	–1.55	–	0.33	–4.38	–1.95	–0.73	–2.56	–5.96	–2.95
LA	–	–	–	NS	–	–	NS	–	–	*	NS	–	–	**
	–	–	–	0.06	–	–	0.38	–	–	–0.52	0.56	–	–	–2.87
DENV+	–	–	NS	–	**	**	–	–	*	***	NS	–	–	NS
	–	–	–0.66	–	–0.82	–1.45	–	–	–3.50	–1.36	–0.60	–	–	2.53
LL × LA	–	–	–	*	–	–	–	–	–	–	–	–	–	–
	–	–	–	1.01	–	–	–	–	–	–	–	–	–	–
LL × DENV+	–	–	–	–	NS	*	–	–	NS	*	–	–	–	–
	–	–	–	–	0.65	1.32	–	–	3.11	1.27	–	–	–	–
LA × DENV+	–	–	–	–	–	–	–	–	–	–	–	–	–	–
	–	–	–	–	–	–	–	–	–	–	–	–	–	–

*Spa* Spaetzle 1 A, *Tol* Toll 1B, *Rel 1* Rel 1 A, *Cac* Cactus, *Dom* Domeless, *Hop* Hopscotch, *PIA* PIAS 2, *Cas* Caspar, *PGRP* PGRP-LC, *Def* Defensin C, *Cec* Cecropin D, *Lyso* Lysozyme, *LL* low larval nutrition, *LA* low adult nutrition, *DENV* +  positive dengue virus infection, *LL × LA, LL × DENV* *+* *, LA × DENV* *+*  the interaction between LL and LA, between LL and DENV + , and between LA and DENV +, *NS* not significant.

*, **, and ***: significant level at 0.05, 0.01, and 0.001. Numbers in each column of the table represent the estimates in each GLM and − represents a predictor is not retained in the best model. Gene expression was measured by the relative fold change of Ct values compared to the internal control gene S7 and reference samples (same nutritional treatments but fed with a noninfectious blood meal) using the delta–delta Ct method.

Collectively, these outcomes suggest a lowered immune responsiveness in mosquitoes that experienced nutritional stress during their larval development. While the impact was less pronounced, adult nutritional stress also downregulated the expression of Imd (Fig. 6j and Table 3) and Lysozyme (Fig. 6n and Table 3). Intriguingly, the downregulation of Domeless (Fig. 6e and Table 3), Hopscotch (Fig. 6f and Table 3), Rel 2 (Fig. 6i and Table 3), and Imd (Fig. 6j and Table 3) was significantly associated with positive infection status (DENV + ). Notably, when interacting with LL (rather than LA), these two factors exhibited a stronger combined effect than their individual effects on the expression of 2 immune genes (Hopscotch and Imd). This suggests that larval nutritional stress can synergistically interfere with the immune response induced by viral exposure. No significant impact of positive infection was observed on the rest of immune genes. The interaction between LL and LA significantly affected Cactus expression, indicating that cross-stage nutritional stress can jointly downregulate Cactus, the negative regulator of the Toll pathway (Fig. 6d and Table 3). There was no significant influence observed from any factors or interactions on the immune genes of Rel 1, PIAS, and Caspar (Fig. 6c, g, h and Table 3).

## Discussion

Our findings demonstrate that nutritional stress, experienced by *Ae. aegypti* mosquitoes during larval and adult stages, detrimentally impacts vital life history traits and anti-DENV immune responses. Particularly, adult nutritional stress and the resulting smaller mosquito size, influenced by larval nutritional stress, amplify the susceptibility of mosquitoes to DENV infection. While previous studies have explored the influence of larval or adult nutrition on individual aspects like life history traits or immunity^29,35,36^, our study provides insight into the combined effects of larval and adult nutrition, shedding light on their interaction’s impact on life history traits, immunity, and infection susceptibility—three key factors in the dynamics of vector-borne pathogen transmission. These revelations enhance our understanding of environmentally mediated effects on vectorial capacity and how environmental management strategies such as source reduction or removal of attractive nectariferous plants might affect pathogen transmission dynamics.

Overall, nutritional stress had an adverse impact on life history traits. Specifically, larval nutritional stress adversely affected adult size, in accordance with prior research^29,35–37^. Furthermore, both larval and adult nutritional stress significantly reduced fecundity. Comparable findings were documented by Vantaux et al.^36^ and Yan et al.^37^, where mosquitoes subjected to lower larval and adult diets produce fewer eggs, implying a direct limitation on egg production due to constrained nutrient and energy supply at each life stage. In line with previous studies^38,39^, ingesting a dengue-infectious blood meal also led to reduced fecundity, pointing to a trade-off between the energy demands for mounting an immune response and reproductive capacity^40^. Our data indicate that the combined impact of these three factors on fecundity is most pronounced with larval and adult nutritional stress, possibly interacting. The influence of DENV exposure on fecundity was more moderate, particularly accentuated only under the conditions of joint larval and adult nutritional stress (indicated by a significant three-way interaction among these variables; Supplementary Fig. S1). It is important to note that the viral titers employed in this experiment were at an intermediate level, and the extent to which higher or lower viral titers could impact the overall effects of viral exposure or the synergistic influence of cross-stage nutritional stress necessitates further exploration. Moreover, our study provided access to blood at a single time point and assessed fecundity based on a single gonotrophic cycle. It is plausible that the effects of DENV exposure could be more pronounced in later life stages. For instance, older females might respond to immune challenges by allocating more resources to reproduction, and the degree to which this response is influenced by diet requires further investigation^41,42^.

Wing length positively influenced fecundity, showing consistency among females reared under both low and normal larval nutrition. Larger individuals tended to lay more eggs, likely due to varying blood intake and conversion efficiency as documented for *Ae. aegypti*^43^. Interestingly, adult nutritional stress alone negatively impacted the presence/absence of eggs, indicating that egg-laying could be exclusively triggered by a certain level of adult energy reserves. Previous studies have suggested that female *Ae. aegypti* that were crowded as larvae require additional nutrition (blood or sucrose) as adults to stimulate primary ovarian follicles development^44^. Alternatively, the timing and size of recent sugar meals could affect fecundity^45^. Whether the low concentration of sucrose in our nutritionally stressed treatment resulted in mosquitoes taking more frequent and larger meals, and whether that affected their subsequent blood meal size (potentially below a critical blood meal size), or whether egg-laying can be resumed when adult energy reserve rises to a normal level in another egg-laying cycle warrants further study. Unexpectedly, larval diet did not significantly affect the probability of laying eggs. In *An. gambiae*, it’s believed that small females often require a second blood meal to complete their first gonotrophic cycle^46^, and similar findings have been reported for *Ae. aegypti*^47^. Additionally, body size of *Ae. aegypti* has further been linked to increased blood-feeding in certain regions^48^. Hence, exploring this finding across diverse genetic backgrounds could yield valuable insights.

Surprisingly, in a multifactorial model emulating the intricate environment, neither larval nor adult nutritional stress independently shortened mosquito survival. However, adult nutritional stress in isolation significantly reduced adult longevity, as evidenced by survival curves, underscoring the pivotal role of adult daily energy supply in promoting longevity. This observation aligns with the findings of Briegel et al.^49^ and Yan et al.^37^, where lower concentrations of sucrose solution led to decreased *Ae. aegypti* longevity. While both positive and negative effects of larval nutritional stress on *Ae. aegypti* survival have been documented in previous studies^10,50^, no significant effect was identified here. Interestingly, the combined impact of larval and adult nutritional stress yielded an interactive reduction in mosquito survival. This significant interaction implies that the effect of adult nutritional stress on survival differed by larval conditions, with more pronounced disparities observed when larvae experienced nutritional deprivation (indicated in Supplementary Fig. S2). Although the ingestion of a dengue-infectious blood meal had no isolated effect, it yielded a substantial impact when analyzed in conjunction with multiple factors and interactions. This suggests that the detrimental effects of DENV infection on survival manifest under conditions of stress. The notable reduction in mosquito survival attributed to the interaction between larval/adult nutritional stress and the ingestion of a dengue-infectious blood meal supports this interpretation. While prior research has reported a negative effect of DENV infection on *Ae. aegypti* survival^38,51^, a number of studies have indicated non-significant effects^52–54^. These conflicting outcomes may stem, in part, from methodological disparities (e.g., different viral strains and titers, larval diets^55,56^), genetic diversity (e.g., different mosquito populations or lab colonies^35,57^), or variations in the mating history of experimental mosquitoes^32^. The three-way interaction among larval and adult nutritional stress and the infectious blood meal highlights that the effect of IB on survival is contingent upon the interplay of nutritional stresses, primarily observed in the context of low sucrose but normal larval nutrition treatment combination (Supplementary Fig. S2). While the exact cause and mechanism behind this finding remain unclear, it is possible that it involves a trade-off with the activation of a more robust immune response.

Our results indicate that adult nutritional stress (LA) increased susceptibility to DENV infection (Fig. 5a), while exerting an overall dampening effect on viral replication in both the bodies and legs of *Ae. aegypti* (Fig. 5c, d), as compared to normal adult nutritional level (NA). However, this dampening effect on viral replication undergoes a reversal after a certain survival time post blood-feeding (~22 dpbf and 34 dpbf for bodies and legs; refer to Fig. 5c, d), attributed to a swifter rise in viral titers at LA compared to NA over the time elapsed since the infectious blood meal. This survival time-dependent influence is mirrored in the modeling of dissemination status, an outcome of augmented viral replication, with a slightly synergistic interaction of LA and survival (see Fig. 5b). These findings highlight how adult energy reserves could potentially shape susceptibility to DENV infection and subsequent viral replication. Although low adult nutrition negatively impacts viral titers per mosquito, prolonged infection duration sees dissemination becoming more pronounced in nutritionally stressed individuals than in those with access to normal nutrition levels. This could hold significant implications for transmission potential, as nutritionally stressed individuals might be more predisposed to transmitting DENV.

Our finding provides novel experimental insights into how adult nutritional stress, stemming from low sugar concentrations, can influence susceptibility to DENV infection and viral replication in mosquitoes. Similarly, Almire et al.^33^ found that feeding on 10% sucrose, compared to no sugar intake, in *Ae. aegypti* appears to block initial gut infection and dissemination, resulting in reduced viral prevalence and intensity of Semliki Forest virus. This might elucidate why mosquitos cultured with a low-quality diet (1% sucrose solution) were more susceptible to infection following an infectious blood meal. Interestingly, these stressed mosquitoes displayed lower mean viral titers (intensity) in their bodies and legs compared to those reared on a normal diet (10% sucrose solution), indicating a potential trade-off between infection prevalence and intensity when facing nutritional constraints. That is, stressed mosquitoes with limited energy supply could be more susceptible to initial infection due to the absence of inhibitory effects conferred by normal-sugar feeding. Yet, the scarcity of energy reserves in these stressed and infected individuals may also hinder substantial replication of DENV. This hypothesis merits further exploration. Alternatively, survival could have significantly influenced viral replication^58^, wherein mosquitoes reared on 1% sucrose solution might have succumbed too early to allow for substantial viral titer increase over time (see Fig. 3c). This could have resulted in fewer mosquitoes from the 1% sugar group surviving longer and developing higher titers. Larval nutritionally stressed mosquitoes (LL) exhibited lower viral prevalence in both bodies and legs compared to their counterparts from a normal larval nutritional level (NL). Our statistical analysis, however, only identified an effect of body size, rather than direct larval nutritional levels, on viral prevalence. The effects of larval nutritional stress on arbovirus infection and dissemination susceptibility in mosquitoes have been well documented, with both significant effects^59–61^ and a lack of effects reported^62,63^. In this study, the food quantity used to induce larval nutritional stress was half that of the normal nutritional level, potentially not being low enough to generate significant infection-related phenotypic disparities. Nonetheless, we find this scenario unlikely, given that the range of sizes is in line with prior experiments on this species (e.g., ref. ^18^). Alternatively, considering that wing length, a measure of adult size and close correlate of larval nutrition, negatively correlates with DENV infection prevalence, the potential impact of larval nutrition on viral prevalence might have indirectly operated through the effect of body size. This indicates that smaller-sized (or larval nutritional-stressed) individuals are more susceptible to DENV infection, consistent with previous studies^18,64,65^.

Our results demonstrate the comprehensive detrimental impact of both larval and adult nutritional stress on the expression of antiviral immune genes, manifested by the downregulation of components across all three immune pathways and their associated antimicrobial peptides. Dietary influence on insect immunity is likely mediated through its effect on immune efficiency^66^, as mounting an immune response entails not only energetic costs^67,68^ but also the utilization of proteins to supply amino acids crucial for constructing immune pathway peptides and effectors like antimicrobial peptides^69,70^. Our bifurcated larval and adult diets confer differential quantities of teneral reserves such as lipids and proteins^43^, as well as distinct carbohydrate quality for mosquitoes. Conceivably, the diminished reserves available under nutritional stress conditions could constrain immune gene expression in our study. This observation resonates with previous research where sugar feeding or higher sugar solution concentrations enhanced mosquito immune responses compared to non-sugar feeding^33,71,72^. Beyond immune gene expression, inadequate carbohydrate supplies may also translate into a decreased expression of reactive oxygen species (ROS) genes^34^, integral to mosquito pathogen defense^26^. However, assessing this aspect was beyond the scope of our current investigation. In accordance with our finding regarding the effect of larval diet, Fellous and Lazzarro^73^ documented stronger immune responses in adult *Drosophila* reared under high-protein larval diets; Banville et al.^74^ revealed reduced immune responses in *Galleria mellonella* resulting from larval food deprivation, implying that protein deficiency during the larval stage could curtail adult immune performance. In contrast to these findings, Telang et al.^29^ reported heightened expression of key immune genes (Spaetzle, Cecropin, and Defensin) in adult *Ae. aegypti* subjected to low larval diets. Notably, this study gauged innate immune expression in mosquitoes devoid of viral challenges. Our results further indicate a significant association between positive DENV infection and the downregulation of four immune genes from pivotal antiviral immune pathways, including JAK-STAT and Imd. While these pathways are acknowledged for being activated in response to arboviral challenges in comparison to unchallenged mosquitoes^22^. our study encompassed all mosquitoes being subjected to a dengue-infectious blood meal. As a result, the potency of the immune response, rather than mere activation, could serve as a determining factor in the outcomes of DENV infection (positive or negative). Interestingly, our analysis did not reveal any association between DENV infection outcomes and Toll pathway activation. This likely signifies that the effects of nutritional stress or the Toll pathway’s involvement in mosquito development exert more pronounced influences, masking DENV infection-responsive induction of this immune pathway. Nonetheless, further studies are required to ascertain any potential interaction between nutrition and Toll pathway-based immunity concerning DENV infection. Antimicrobial effectors like Cecropin and Defensin are pivotal constituents of the antimicrobial response regulated by Toll and Imd pathways. However, our data evince no induction of gene expression in these effectors by DENV infection or its interaction with nutritional stress. A possible explanation is that these effectors are upregulated in response to DENV exposure, but that infection outcomes are a result of broader interactions that we were not able to distinguish here. Toll, JAK-STAT, and Imd are recognized immune pathways constituting the antiviral defense repertoire of mosquitoes, including *Ae. aegypti*^24–26,29,75^. Our findings align with other insects where nutrition prominently factors in mounting an effective immune response^76–79^. In addition, our results imply that mosquito immune gene expression is substantially shaped by larval nutrition, spotlighting the “carry-over effects” of larval ecology on mosquito immunity, even though we didn’t measure reactive oxygen species or the expression of genes related to ROS production.

Our study has several limitations. For instance, we used a F_19_ generation of *Ae. aegypti* mosquitoes from a laboratory colony established from eggs collected in Key West, FL and the P-84 strain of DENV-4. To enhance the generalizability of our findings, investigations involving F_1_ generations of various populations exposed to viral stocks recently isolated from the same regions could provide valuable insights. In addition, the viral titer used for challenging mosquitoes in our study was maintained at an intermediate level. The impact on life history traits, vector competence, and immunity when using higher or lower viral titers remains unclear and necessitates further inquiry. In an effort to streamline experimental design, we restricted larval and adult nutrition to two levels and allowed mosquitoes to consume a single full-blood meal. However, under natural conditions, female *Ae. aegypti* feed on blood every few days. It remains uncertain whether nutritional stress due to limited sugar access would yield similar outcomes when ample blood-feeding opportunities are present or if variations in blood host availability, in the absence of sugar sources, would produce comparable effects on life history traits and vectorial capacity. These queries necessitate future investigation. Given the possible divergences in vector competence and immunity between laboratory and wild mosquitoes, as well as the substantial diversity in wild larval and adult food availability and quality, and the multiple and partial blood-feeding characteristic of field-caught blood-fed mosquitoes, our findings may not precisely reflect the infection and immune outcomes under nutritional stress and viral challenge in field conditions. Furthermore, we did not assess whether mosquitoes exposed to low levels of adult nutrition could compensate by consuming larger or more frequent meals, a facet that future research could directly address. Nonetheless, prior studies investigating the effects of sugar feeding on mosquito life history traits or vector competence, which featured comparable or even smaller differences in sugar concentration, reported significant outcomes attributed to variations in sugar concentration (e.g., Lambrechts et al.^80^; Vaidyanathan et al.^81^; Lalubin et al.^82^; Sangare et al.^83^). Based on the effects of adult nutrition observed in our study, it appears that compensatory feeding, if present, was insufficient to negate the disparities in nutritional levels. We used multiple rearing trays or mosquito-keeping cages for each treatment level during larval and adult rearing, as well as during blood-feeding, to ensure an adequate number of experimental mosquitoes underwent the winnowing process from larvae to engorged adults. Although this approach could theoretically introduce unforeseen variability among trays/cages, potentially leading to concerns of pseudo-replication if observed patterns were primarily influenced by such variations rather than treatment effects, we find this likelihood to be minimal in our case. This is because potential factors contributing to variations among trays/cages were effectively standardized or randomized. To explore the potential impact of DENV ingestion on mosquito lifespan and the potential association between DENV infection outcomes and mosquito lifespan, we conducted the DENV infection assay upon the death of mosquitoes in the survival assay, which we manually observed twice daily. However, delayed detection of deceased mosquitoes within the 8-h observation interval could have led to diminished titers in the subsequent DENV assay due to a certain degree of RNA degradation, especially if the delayed detection events were unevenly distributed across treatment levels. Future studies might mitigate this potential bias by shortening the observational interval while maintaining manageable labor intensity and without disturbing resting mosquitoes.

Comprehending the impact of nutrition on phenotypic traits in medically significant species holds paramount importance. Our study, in its entirety, unravels the intricate nexus between nutrition and life history traits, susceptibility to DENV infection, and the expression of immune genes in *Ae. aegypti*, intricately linked to the vectorial capacity of this pivotal arbovirus vector. Future investigations should expand upon these vectorial capacity-associated traits, encompassing critical aspects such as the effects on the extrinsic incubation period^84^. While reduced larval nutrition in malaria mosquitoes has been demonstrated to prolong the development of *P. falciparum*^30^, the interplay between larval and adult nutrition and its impact on this trait remain unexplored. It’s noteworthy that vector competence is commonly assessed in laboratory settings where females often experience consistent larval and adult nutrition levels. However, in natural environments, larvae often face stressors including inadequate nutrition and fierce competition, which may lead to the emergence of smaller adults^85^. Hence, our findings shed light on overlooked variables that can introduce variability into laboratory-based experiments’ outcomes. The debate regarding the extent to which *Ae. aegypti* rely on plant sugars for energy reserves remains ongoing and may hinge on their habitats^86–89^. Nevertheless, our results underscore the pivotal role of low sugar intake in enhancing susceptibility to DENV infection. This insight holds significance in scenarios where anthropophilic *Ae. aegypti* rarely feed on sugar, potentially amplifying the transmission efficiency of arboviruses. Our study deepens our understanding of the environmental elements that modulate vectorial capacity, carrying enriching implications for strategies in vector-borne disease control through environmental management (e.g., targeting invasive plant species or larval sources). These strategies could serve to mitigate the transmission of arboviruses effectively.

## Methods

### Mosquitoes and nutritional treatments

F_19_ generation of *Ae. aegypti* mosquitoes from a laboratory colony established from eggs collected in Key West, FL were reared following a standard protocol previously described^32^. Eggs were provisioned with a pinch (~1 g) of brain heart infusion, after which 1st instar larvae were transferred to identical larval trays, with 100 larvae in 1 L of deionized water per tray. The larvae were randomly assigned to two levels of larval nutritional treatment^29,50^ (Fig. 1): a well-nourished level where 100 mg of rabbit chow: lactalbumin: yeast (1:1:1) diet was provided on days 2, 4, 5, and 6 after hatching, representing normal larval nutrition (NL); or a malnourished level where 100 mg of the same diet was provided only on days 2 and 6 after hatching, representing low larval nutrition (LL). Adults eclosed on days 7–9 post hatching at each level of the larval nutritional treatment were randomly assigned to either level of adult nutritional treatment with different food quality (Fig. 1): a well-nourished level with ad libitum access to a 10% sucrose solution, representing normal adult nutrition (NA); or a malnourished level with ad libitum access to 1% sucrose solution, representing low adult nutrition (LA). Over a span of 3–5 days, males and females were cohabited to facilitate mating. Throughout the experimentation period, larvae and adults were maintained under consistent conditions of 27 (±1) °C temperature, 75 (±5)% relative humidity, and a 12-h light/dark cycle. The nutritional treatments were executed in two successive blocks, wherein each treatment from either block accounted for half of the total sample size.

### Infectious blood meals and blood-feeding treatment

African green monkey kidney cells (hereafter “Vero”, CCL-81, ATCC, USA) were cultured in L-15 media supplemented with 10% fetal bovine serum (FBS), 1% penicillin-streptomycin, and 1% amino acids (hereafter “growth media”) at 37 °C with 5% CO_2_. The P-84 strain of DENV-4 was propagated in Vero cells according to a standard protocol:^90^ 0.25 ml of DENV stock containing growth media was used to infect confluent monolayer Vero cells in 25-cm^2^ flasks. After 1 hr of incubation, 5 ml of L-15 media was added to each flask for a final volume of 5.25 ml per flask and incubated for 5 days. Infected cells and growth media were harvested and mixed with citrated bovine blood (BBC050, HemoStat Laboratories, USA) in a 1:1 ratio and used as infectious blood meal. Uninfected Vero cells and growth media were maintained, harvested, and mixed with bovine blood under the same conditions, which served as the control (noninfectious blood meal). Eight-to 10-day-old *Ae. aegypti* females that had gone through the above nutritional treatments were sugar-starved for 24 h and randomly offered either an infectious blood meal (IB) or a noninfectious blood meal (NB) using a membrane feeding system for 45 min (Fig. 1). During this blood-feeding process, an equal number of starved females were placed in same cages, and each treatment level consisted of two cages. Aliquots of infectious blood meals were taken just before and after blood-feeding and stored at −80 °C for viral titration. The blood-feeding treatment was performed in two subsequent blocks.

### Measurements of life history traits

After offering blood meals (IB or NB), mosquitoes from two blood-feeding cages within each treatment level were simultaneously cold-anesthetized at 4 °C. They were then mixed up on a chill table to randomly select around 50 fully engorged individuals per treatment level per block. Subsequently, the chosen females were individually placed in new 8 oz. paperboard containers with lids and small oviposition cups. The cups and individual specimens were placed on a tray, and both the location on the tray and the shelf in the incubator in which the tray was placed was rotated daily. The blood-fed female mosquitoes were maintained under the same conditions and nutritional levels as before until death. Mortality of blood-fed mosquitoes was checked twice per day at 9 am and 5 pm and survival was measured as the number of days from blood-feeding to death. Once a dead mosquito was observed, all the eggs inside a cup were counted using a stereomicroscope, which was used as a surrogate for fecundity of a mosquito^91^. Mosquito samples were immediately stored at −80 °C for follow-up dissection and DENV detection^38^. Wings were dissected on dry ice and wing length was measured as the length between the axial incision and the apical margin excluding the fringe of scales^92^, as a surrogate for body size. The wing length measurement was performed with an inverted microscope (IX51, Olympus, Japan) and Olympus cellSens Entry 2.3 software. Samples for life history trait measurements were taken from the two blocks equally.

### Assay for infection and dissemination outcome

Following the measurement of life history traits, the mosquitoes that fed on IB were immediately dissected on dry ice and their bodies and legs were used to test infection and dissemination outcome, respectively. Total RNA was extracted from mosquito bodies and legs, respectively, using QIAamp Virus BioRobot MDx Kit according to the manufacturer’s protocol in a BioRobot® Universal System (Qiagen, Hilden, Germany). Quantitative real-time RT-PCR (hereafter ‘RT-qPCR’) was performed using the Taqman™ Fast Virus 1-Step Master Mix (Thermo Fisher, Waltham, USA) and the QuantStudio™ 5 Real Time PCR System (Thermo Fisher, Waltham, USA). Dengue-4 viral status (positive or negative) were determined based on a cutoff crossing threshold (Ct) value of 37.00 recommended by CDC^93^ using the primers (forward: TTGTCCTAATGATGCTGGTCG; reverse: TCCACCTGAGACTCCTTCCA) and probe (Joe-TTCCTACTCCTACGCATCGCATTCCG-BHQ) from Johnson et al.^94^. Dengue-4 viral titer was quantified as DENV copy number per tissue using RT-qPCR^95^. Briefly, a 1991 bp (insertion sequence: 5’–TTG TCC TAA TGA TGC TGG TCG CCC CAT CTT ACG GAA TGC GAT GCG TAG GAG TAG GAA ACA GAG ACT TTG TGG AAG GAG TCT CAG GTG GA–3’) DENV-4 minigene (IDT, Coralville, USA) was serially diluted by 10-fold and run in RT-qPCR in triplicate to generate a standard curve; and mosquito samples were tested in RT-qPCR and their Ct values were converted to viral titers according to the standard curve^96,97^. Numeric data for infection and dissemination outcome are presented in Supplementary Table S1. Samples for the assay were taken from the two blocks equally.

### Assay for immune gene expression

An additional 36 blood-fed mosquitoes that had gone through the aforementioned nutritional and blood-feeding treatments were used for the immune gene expression assay (Fig. 1). Ten days after blood-feeding, these mosquitoes were frozen and stored at −80 °C for further processing. Ten days were selected as the time when disseminating infection could best be assessed in all treatment groups. RNA extraction, cDNA synthesis and real-time PCR were performed as follows: total RNA was extracted from the mosquito carcass by following the manufacturer’s protocol using a NucleoSpin® RNA isolation Kit (Macherey-Nagel, Düren, Germany); cDNA transcript was synthesized using PrimeScript™ RT reagent Kit with genomic DNA eraser (Takara-Bio, Shiga, Japan); real-time PCR was performed using the SensiFAST™ SYBR® Hi-ROX One-Step Kit (Bioline, London, UK) and the QuantStudio™ 5 Real Time PCR System (Thermo Fisher, Waltham, USA). Twelve mosquitoes that had fed on IB and became infected with DENV (3 positive for each IB group) and another 12 mosquitoes that had fed on IB but not infected with DENV (3 negative for each IB group) were used as the experimental groups, and the remaining 12 that had fed on NB (3 for each NB group) were used as the control groups (Fig. 1). Each NB group was used as the unique control for the IB group (3 positive and 3 negative) that had received the same larval and adult nutritional treatments (i.e., each pair of NB-IB within the same brackets of Fig. 1). Therefore, three independent biological replicates for each group were conducted and all PCR reactions were performed in technical triplicates. The ribosomal protein S7 gene was used for normalization of cDNA templates. Relative fold changes in the expression of 14 immune genes belonging to the Toll, JAK-STAT, and Imd pathways as well as antimicrobial peptides were calculated following the 2^− ΔΔCT^ method^98^. All primers used for real-time PCR are presented in Supplementary Table S2. Quantitative gene expression data are presented in Supplementary Table S3. Samples for the assay were taken from the second block exclusively.

### DENV-4 titers in infectious blood meals

Viral titers in the infectious blood meals were measured by both focus forming assay (FFA) and RT-qPCR. FFA was performed following a protocol modified from ref. ^99^: briefly, the aliquots of infectious blood meal reserved before and after blood-feeding were serially diluted and inoculated into confluent monolayer Vero cells in 96-well plates. After incubation for 48 h at 37 °C and 5% CO_2_, the plates were peroxidase-immunostained using a mouse monoclonal antibody against DENV-4 (MAb 4G2) and a goat anti-mouse HRP conjugate as the primary and secondary antibody, respectively. RT-qPCR for viral titers in the same aliquots of infectious blood meals were performed with the methods mentioned above in the assay for infection and dissemination outcome. The averaged viral titer in infectious blood meals used before and after blood-feeding was 1.21 × 10^4^ focus forming units/ml and 8.29 log 10 total viral particles/ml in FFA and RT-qPCR, respectively.

### Statistics and reproducibility

GLMs with Gaussian distribution were fitted to test if there are significant differences in wing length between nutritional treatments and their interactions. To accommodate for zero values in fecundity, the probability of laying eggs or not (presence/absence) was considered in the binomial part while the number of eggs laid by mosquitoes (excluding non-egg layers) was considered in the Poisson part of hurdle models, where the effects of nutritional treatments, blood-feeding treatment, wing length, and their interactions on mosquito fecundity were assessed, using the R package *pscl*^100^. Survival analyses were performed to test the effects of nutritional treatments, blood-feeding treatment, wing length, and their interactions on death risk of *Ae. aegypti* using the CPH model and the survival probabilities of *Ae. aegypti* between different levels of the above three treatments using the packages *survival*^101^ and *survminer*^102^ with Kaplan–Meier method and Log-Rank test. GLMs with binomial error and logit link function were performed to test the variations in dengue viral status (positive or negative) with nutritional treatments, wing length, survival (i.e., time since the normal/infectious blood meal), and their interactions as explanatory variables. The potential effects of these variables on dengue viral titers were also assessed in GLMs with Gaussian distribution. To assess the impact of nutritional treatments and infection status on immune gene expression, GLMs were performed using relative fold change in the expression of each gene as the response and larval nutrition, adult nutrition, infection status and their interactions as predictors. Block (I or II) was also included as a fixed effect (blocking variable) into all the above models except GLMs for gene expression assay (done in one block). Before fitting linear models, the normality of the responses (wing length, viral titers and relative fold change) was examined, and the homoscedasticity of residuals was also inspected after fitting these models. Model selection for the best-fitted hurdle model, CPH model, and GLM was based on the lowest values of Akaike information criterion (AIC) or corrected AIC for small sample size (AICc)^103^. Statistical analyses were carried out in R software v. 4.3.1^104^. A list of models used in the statistical analyses is presented in Supplementary Table S4.

### Reporting summary

Further information on research design is available in the Nature Portfolio Reporting Summary linked to this article.

### Supplementary information

Peer Review File

Supplementary Information

Description of Additional Supplementary Files

Supplementary Data 1

Reporting Summary

## Data Availability

All data supporting the findings of this study are available within the paper and its Supporting Information. Life history trait data for all experimental mosquitoes are provided in Supplementary Data 1.
