# Supplementary material for: Nutritional stress compromises mosquito fitness and antiviral immunity, while enhancing dengue virus infection susceptibility
Source: Commun Biol. 2023 Nov 6;6:1123. doi: 10.1038/s42003-023-05516-4 (PMC10628303; doi:10.1038/s42003-023-05516-4)
Supplement: Supplementary file 2 — Supplementary Information [file 42003_2023_5516_MOESM2_ESM.pdf]

communications biology

Supporting Information for

Nutritional stress compromises mosquito fitness and antiviral immunity, while enhancing dengue virus infection susceptibility

Jiayue Yan[1]*, Chang-Hyun Kim[1], Leta Chesser[1], Jose L. Ramirez[2] and Chris M. Stone[1]

* Corresponding author:

Email: jiayue@illinois.edu

This PDF file includes:

Figures S1 to S2

Tables S1 to S4

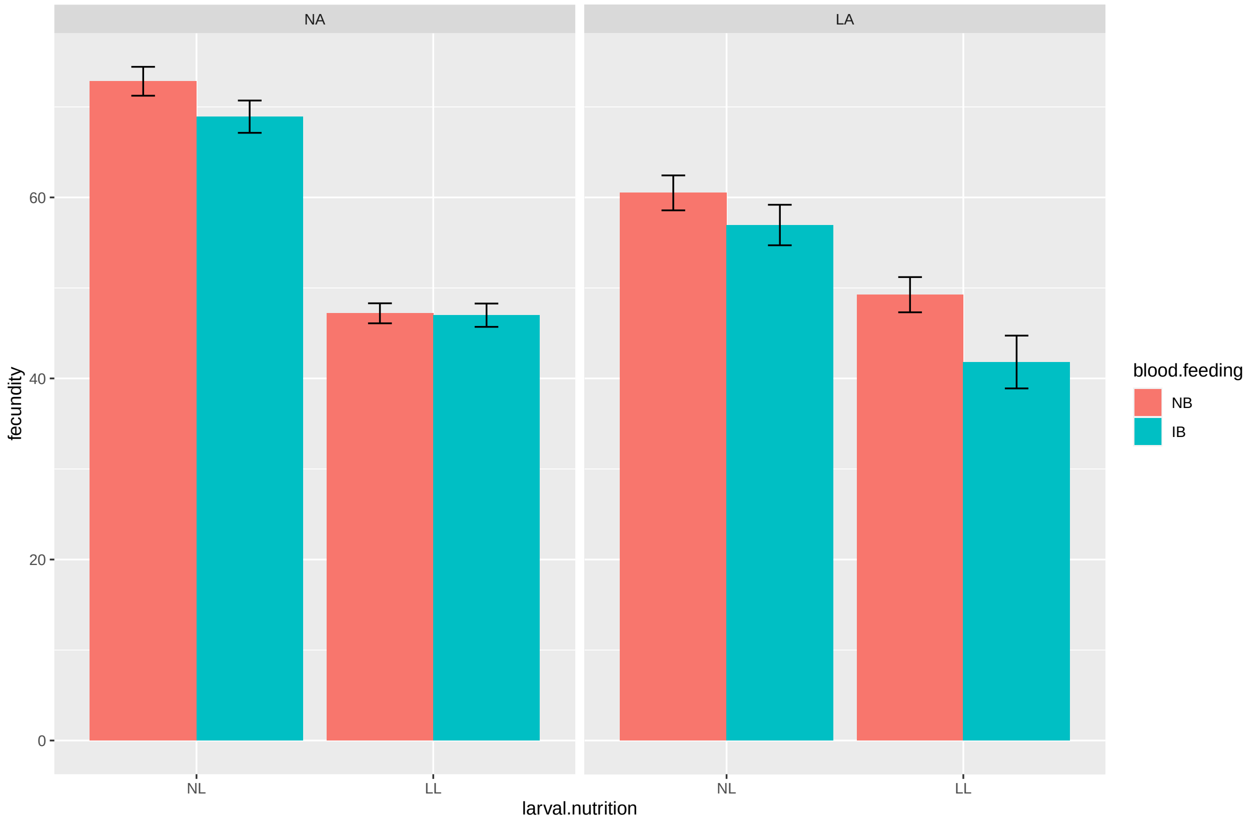

Fig. S1. Fecundity of *Ae. aegypti* reared at normal/low larval/adult nutritional levels (referred to as 'NL', 'LL', 'NA', 'LA') and subsequently exposed to a non-infectious/dengue infectious blood meal (referred to as 'NB', 'IB'). Fecundity is represented as the mean ± standard error (SE) count of mosquito eggs.

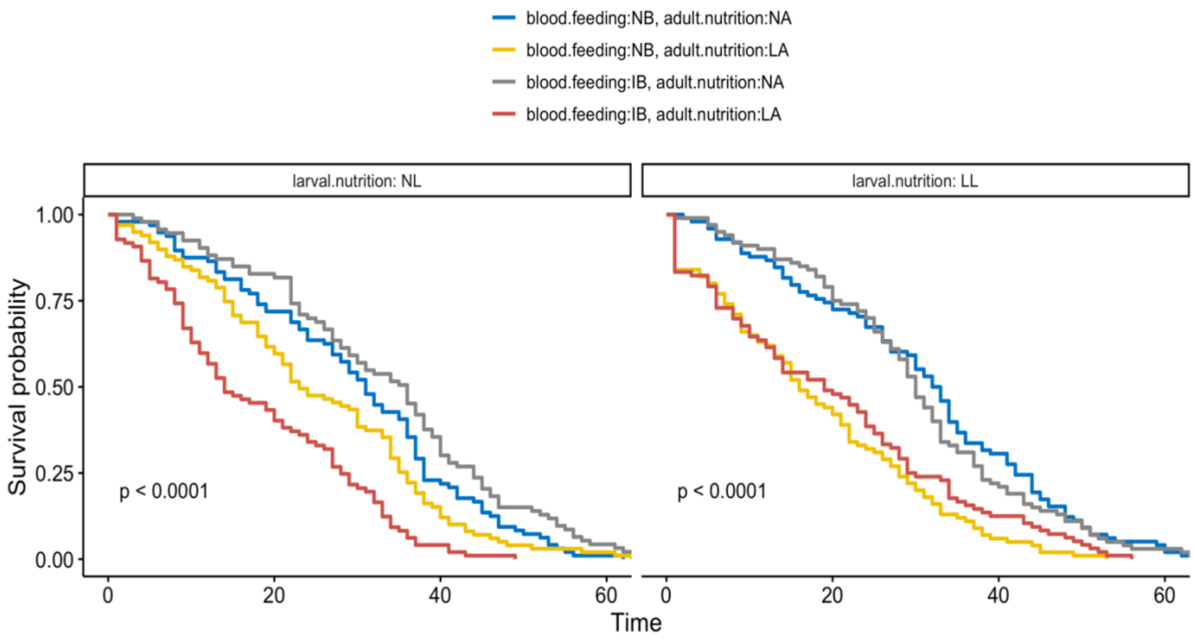

Fig. S2. Survival probabilities of *Ae. aegypti* across distinct larval and adult nutritional treatments and blood feeding sources. NL: normal larval nutrition, LL: low larval nutrition, NA: normal adult nutrition, LA: low adult nutrition, NB: non-infectious blood meal, IB: dengue infectious blood meal. There was an overall significant difference between NA and LA in the survival curves from the Cox Proportional-Hazards model.

Table S1. Dengue viral status and titer in *Aedes aegypti*. LL: low larval nutrition, NL: normal larval nutrition, LA: low adult nutrition, NA: normal adult nutrition, N/A: not available. Infection and dissemination status were determined by a cutoff Ct value of 37.00. Infection and dissemination titers were log 10 transformed.

ID	Block	Larval nutrition	Adult nutrition	Infection status	Dissemination status	Infection titer (log 10)	Dissemination titer (log 10)
1	I	LL	LA	Positive	Positive	6.376563727	4.826895641
2	I	LL	LA	Negative	Negative	N/A	N/A
3	I	LL	LA	Negative	Negative	N/A	N/A
4	I	LL	LA	Positive	Positive	7.3248271	5.884049317
5	I	LL	LA	Positive	Positive	6.828611139	5.466362122
6	I	LL	LA	Negative	Negative	N/A	N/A
7	I	LL	LA	Negative	Negative	N/A	N/A
8	I	LL	LA	Positive	Positive	6.816241552	5.74034929
9	I	LL	LA	Negative	Negative	N/A	N/A
10	I	LL	LA	Positive	Negative	5.713247127	N/A
11	I	LL	LA	Positive	Positive	7.11359276	5.524479859
12	I	LL	LA	Positive	Positive	6.923599386	4.911124357
13	I	LL	LA	Positive	Positive	7.272887972	4.749688221
14	I	LL	LA	Negative	Negative	N/A	N/A
15	I	LL	LA	Positive	Negative	6.257096879	N/A
16	I	LL	LA	Negative	Negative	N/A	N/A
17	I	LL	LA	Positive	Negative	4.619451709	N/A
18	I	LL	LA	Positive	Positive	6.47926783	5.84246433
19	I	LL	LA	Positive	Positive	6.843958862	5.884941705
20	I	LL	LA	Negative	Negative	N/A	N/A
21	I	LL	LA	Negative	Negative	N/A	N/A
22	I	LL	LA	Positive	Positive	6.917689712	5.429176952
23	I	LL	LA	Negative	Negative	N/A	N/A
24	I	LL	LA	Negative	Negative	N/A	N/A
25	I	LL	LA	Positive	Positive	6.340395859	5.338756151
26	I	LL	LA	Positive	Positive	5.855182916	2.665104133
27	I	LL	LA	Positive	Positive	2.97809229	1.729960103
28	I	LL	LA	Negative	Negative	N/A	N/A
29	I	LL	LA	Positive	Positive	7.317099759	5.871140102
30	I	LL	LA	Negative	Negative	N/A	N/A
31	I	LL	LA	Negative	Negative	N/A	N/A
32	I	LL	LA	Positive	Positive	5.967511408	4.811354956
33	I	LL	LA	Positive	Positive	7.192094669	5.951717514
34	I	LL	LA	Positive	Positive	7.164991293	5.697615499

35	I	LL	LA	Positive	Positive	7.026751425	5.997683191
36	I	LL	LA	Negative	Negative	N/A	N/A
37	I	LL	LA	Positive	Positive	7.026329707	5.614914041
38	I	LL	LA	Positive	Negative	5.959935912	N/A
39	I	LL	LA	Positive	Negative	4.446868662	N/A
40	I	LL	LA	Positive	Positive	7.431824256	5.011391665
41	I	LL	LA	Positive	Negative	1.91395213	N/A
42	I	LL	LA	Positive	Positive	7.446571525	6.027942079
43	I	LL	LA	Positive	Negative	6.789371414	N/A
44	I	LL	LA	Negative	Negative	N/A	N/A
45	I	LL	LA	Negative	Negative	N/A	N/A
46	I	LL	LA	Positive	Positive	6.872837582	5.959716616
47	I	LL	LA	Positive	Positive	6.847654462	2.589195341
48	I	LL	LA	Negative	Negative	N/A	N/A
49	II	LL	LA	Positive	Positive	3.359309203	1.569829434
50	II	LL	LA	Positive	Negative	4.924760538	N/A
51	II	LL	LA	Negative	Negative	N/A	N/A
52	II	LL	LA	Positive	Positive	7.024232074	5.571421119
53	II	LL	LA	Positive	Positive	4.510387043	1.27495198
54	II	LL	LA	Negative	Negative	N/A	N/A
55	II	LL	LA	Positive	Negative	6.221116572	N/A
56	II	LL	LA	Positive	Positive	8.079542587	6.27940962
57	II	LL	LA	Negative	Negative	N/A	N/A
58	II	LL	LA	Negative	Negative	N/A	N/A
59	II	LL	LA	Negative	Negative	N/A	N/A
60	II	LL	LA	Positive	Negative	2.007278593	N/A
61	II	LL	LA	Negative	Negative	N/A	N/A
62	II	LL	LA	Positive	Positive	4.514822135	3.00053768
63	II	LL	LA	Negative	Negative	N/A	N/A
64	II	LL	LA	Negative	Negative	N/A	N/A
65	II	LL	LA	Negative	Negative	N/A	N/A
66	II	LL	LA	Negative	Negative	N/A	N/A
67	II	LL	LA	Negative	Negative	N/A	N/A
68	II	LL	LA	Positive	Negative	4.987821898	N/A
69	II	LL	LA	Positive	Positive	3.884220957	1.606775066
70	II	LL	LA	Positive	Positive	4.39652776	2.95487605
71	II	LL	LA	Positive	Positive	7.001346559	5.555628556
72	II	LL	LA	Negative	Negative	N/A	N/A
73	II	LL	LA	Negative	Negative	N/A	N/A
74	II	LL	LA	Positive	Negative	5.660130195	N/A

75	II	LL	LA	Positive	Negative	5.283159106	N/A
76	II	LL	LA	Positive	Positive	4.376617366	3.389998427
77	II	LL	LA	Positive	Positive	7.227228237	5.47454026
78	II	LL	LA	Positive	Positive	6.749609515	5.497812261
79	II	LL	LA	Negative	Negative	N/A	N/A
80	II	LL	LA	Positive	Negative	4.453044189	N/A
81	II	LL	LA	Positive	Positive	7.028307243	4.860961647
82	II	LL	LA	Negative	Negative	N/A	N/A
83	II	LL	LA	Positive	Positive	3.651779871	2.579036537
84	II	LL	LA	Negative	Negative	N/A	N/A
85	II	LL	LA	Negative	Negative	N/A	N/A
86	II	LL	LA	Positive	Negative	6.073822436	N/A
87	II	LL	LA	Positive	Positive	4.555501304	1.800606042
88	II	LL	LA	Positive	Negative	4.455659578	N/A
89	II	LL	LA	Positive	Negative	4.230946187	N/A
90	II	LL	LA	Positive	Negative	5.952650868	N/A
91	II	LL	LA	Negative	Negative	N/A	N/A
92	II	LL	LA	Positive	Negative	3.058767779	N/A
93	II	LL	LA	Positive	Positive	5.821226163	1.258238542
94	II	LL	LA	Positive	Positive	6.970437909	5.684927905
95	II	LL	LA	Negative	Negative	N/A	N/A
96	II	LL	LA	Positive	Negative	4.57668979	N/A
97	I	NL	LA	Negative	Negative	N/A	N/A
98	I	NL	LA	Positive	Positive	7.061424687	6.374692261
99	I	NL	LA	Positive	Positive	4.641730863	2.278541158
100	I	NL	LA	Negative	Negative	N/A	N/A
101	I	NL	LA	Negative	Negative	N/A	N/A
102	I	NL	LA	Positive	Positive	3.750415675	1.316073195
103	I	NL	LA	Negative	Negative	N/A	N/A
104	I	NL	LA	Positive	Negative	1.984760821	N/A
105	I	NL	LA	Positive	Positive	7.469520229	4.939832323
106	I	NL	LA	Positive	Positive	4.870852435	3.259452021
107	I	NL	LA	Positive	Negative	2.204170654	N/A
108	I	NL	LA	Positive	Positive	6.637249936	3.148220184
109	I	NL	LA	Positive	Positive	7.518563077	6.296186961
110	I	NL	LA	Positive	Negative	7.017150205	N/A
111	I	NL	LA	Negative	Negative	N/A	N/A
112	I	NL	LA	Negative	Negative	N/A	N/A
113	I	NL	LA	Positive	Positive	7.07576924	4.836526052
114	I	NL	LA	Positive	Positive	7.467203791	5.869842095

115	I	NL	LA	Positive	Positive	7.049508128	5.538720763
116	I	NL	LA	Positive	Positive	6.834637352	3.757120502
117	I	NL	LA	Positive	Positive	6.111582007	4.559224059
118	I	NL	LA	Negative	Negative	N/A	N/A
119	I	NL	LA	Positive	Positive	6.834468506	3.757782437
120	I	NL	LA	Positive	Positive	7.733120851	6.141622638
121	I	NL	LA	Negative	Negative	N/A	N/A
122	I	NL	LA	Positive	Positive	7.384543388	5.843605126
123	I	NL	LA	Negative	Negative	N/A	N/A
124	I	NL	LA	Positive	Positive	7.460057861	5.846062115
125	I	NL	LA	Positive	Negative	2.451583643	N/A
126	I	NL	LA	Positive	Positive	7.177115802	6.205616584
127	I	NL	LA	Positive	Positive	4.15796464	1.783910367
128	I	NL	LA	Positive	Positive	5.638440344	2.955247677
129	I	NL	LA	Negative	Negative	N/A	N/A
130	I	NL	LA	Negative	Negative	N/A	N/A
131	I	NL	LA	Negative	Negative	N/A	N/A
132	I	NL	LA	Positive	Negative	4.646846872	N/A
133	I	NL	LA	Negative	Negative	N/A	N/A
134	I	NL	LA	Negative	Negative	N/A	N/A
135	I	NL	LA	Negative	Negative	N/A	N/A
136	I	NL	LA	Negative	Negative	N/A	N/A
137	I	NL	LA	Negative	Negative	N/A	N/A
138	I	NL	LA	Negative	Negative	N/A	N/A
139	I	NL	LA	Negative	Negative	N/A	N/A
140	I	NL	LA	Negative	Negative	N/A	N/A
141	I	NL	LA	Negative	Negative	N/A	N/A
142	I	NL	LA	Negative	Negative	N/A	N/A
143	I	NL	LA	Negative	Negative	N/A	N/A
144	I	NL	LA	Negative	Negative	N/A	N/A
145	II	NL	LA	Negative	Negative	N/A	N/A
146	II	NL	LA	Negative	Negative	N/A	N/A
147	II	NL	LA	Negative	Negative	N/A	N/A
148	II	NL	LA	Positive	Positive	6.422579038	1.561691189
149	II	NL	LA	Negative	Negative	N/A	N/A
150	II	NL	LA	Negative	Negative	N/A	N/A
151	II	NL	LA	Negative	Negative	N/A	N/A
152	II	NL	LA	Positive	Positive	6.992589536	1.620908699
153	II	NL	LA	Positive	Negative	6.179583329	N/A
154	II	NL	LA	Negative	Negative	N/A	N/A

155	II	NL	LA	Positive	Positive	4.239648427	1.2483291
156	II	NL	LA	Positive	Positive	7.588755519	4.458082087
157	II	NL	LA	Positive	Negative	5.906028887	N/A
158	II	NL	LA	Positive	Negative	6.203421764	N/A
159	II	NL	LA	Positive	Negative	5.048883361	N/A
160	II	NL	LA	Positive	Positive	7.228984402	5.224840391
161	II	NL	LA	Negative	Negative	N/A	N/A
162	II	NL	LA	Positive	Positive	7.404386347	6.01875282
163	II	NL	LA	Positive	Positive	7.447366561	5.484589432
164	II	NL	LA	Negative	Negative	N/A	N/A
165	II	NL	LA	Negative	Negative	N/A	N/A
166	II	NL	LA	Positive	Positive	6.573465967	5.094322525
167	II	NL	LA	Negative	Negative	N/A	N/A
168	II	NL	LA	Positive	Positive	7.724399991	6.014207307
169	II	NL	LA	Negative	Negative	N/A	N/A
170	II	NL	LA	Negative	Negative	N/A	N/A
171	II	NL	LA	Negative	Negative	N/A	N/A
172	II	NL	LA	Negative	Negative	N/A	N/A
173	II	NL	LA	Positive	Positive	6.703947152	5.609349449
174	II	NL	LA	Positive	Positive	7.008934041	6.265252465
175	II	NL	LA	Negative	Negative	N/A	N/A
176	II	NL	LA	Positive	Positive	6.614015386	1.363317881
177	II	NL	LA	Positive	Negative	5.647304277	N/A
178	II	NL	LA	Positive	Positive	5.294156867	2.71334877
179	II	NL	LA	Negative	Negative	N/A	N/A
180	II	NL	LA	Negative	Negative	N/A	N/A
181	II	NL	LA	Positive	Negative	7.555413547	N/A
182	II	NL	LA	Negative	Negative	N/A	N/A
183	II	NL	LA	Negative	Negative	N/A	N/A
184	II	NL	LA	Negative	Negative	N/A	N/A
185	II	NL	LA	Positive	Negative	4.56581133	N/A
186	II	NL	LA	Positive	Positive	6.022612244	5.61488756
187	II	NL	LA	Negative	Negative	N/A	N/A
188	II	NL	LA	Negative	Negative	N/A	N/A
189	II	NL	LA	Positive	Positive	5.889982488	5.22881657
190	II	NL	LA	Positive	Negative	6.209401578	N/A
191	II	NL	LA	Negative	Negative	N/A	N/A
192	II	NL	LA	Negative	Negative	N/A	N/A
193	II	NL	LA	Positive	Negative	4.92631221	N/A
194	I	LL	NA	Negative	Negative	N/A	N/A

195	I	LL	NA	Negative	Negative	N/A	N/A
196	I	LL	NA	Negative	Negative	N/A	N/A
197	I	LL	NA	Positive	Negative	0.063289407	N/A
198	I	LL	NA	Negative	Negative	N/A	N/A
199	I	LL	NA	Positive	Negative	5.557224895	N/A
200	I	LL	NA	Negative	Negative	N/A	N/A
201	I	LL	NA	Positive	Positive	6.001489102	5.526196351
202	I	LL	NA	Negative	Negative	N/A	N/A
203	I	LL	NA	Negative	Negative	N/A	N/A
204	I	LL	NA	Negative	Negative	N/A	N/A
205	I	LL	NA	Negative	Negative	N/A	N/A
206	I	LL	NA	Negative	Negative	N/A	N/A
207	I	LL	NA	Positive	Positive	6.972885609	5.915304347
208	I	LL	NA	Positive	Positive	6.523895489	5.467509953
209	I	LL	NA	Positive	Negative	6.323211561	N/A
210	I	LL	NA	Positive	Negative	1.312911463	N/A
211	I	LL	NA	Negative	Negative	N/A	N/A
212	I	LL	NA	Negative	Negative	N/A	N/A
213	I	LL	NA	Negative	Negative	N/A	N/A
214	I	LL	NA	Negative	Negative	N/A	N/A
215	I	LL	NA	Positive	Positive	6.436909472	3.868883476
216	I	LL	NA	Positive	Positive	1.807563335	1.817283513
217	I	LL	NA	Negative	Negative	N/A	N/A
218	I	LL	NA	Positive	Positive	6.827561189	3.633104719
219	I	LL	NA	Positive	Negative	6.399375358	N/A
220	I	LL	NA	Negative	Negative	N/A	N/A
221	I	LL	NA	Negative	Negative	N/A	N/A
222	I	LL	NA	Negative	Negative	N/A	N/A
223	I	LL	NA	Negative	Negative	N/A	N/A
224	I	LL	NA	Negative	Negative	N/A	N/A
225	I	LL	NA	Negative	Negative	N/A	N/A
226	I	LL	NA	Positive	Positive	6.089494196	5.841101485
227	I	LL	NA	Negative	Negative	N/A	N/A
228	I	LL	NA	Negative	Negative	N/A	N/A
229	I	LL	NA	Negative	Negative	N/A	N/A
230	I	LL	NA	Negative	Negative	N/A	N/A
231	I	LL	NA	Negative	Negative	N/A	N/A
232	I	LL	NA	Negative	Negative	N/A	N/A
233	I	LL	NA	Negative	Negative	N/A	N/A
234	I	LL	NA	Negative	Negative	N/A	N/A

235	I	LL	NA	Negative	Negative	N/A	N/A
236	I	LL	NA	Negative	Negative	N/A	N/A
237	I	LL	NA	Negative	Negative	N/A	N/A
238	I	LL	NA	Positive	Negative	4.887472418	N/A
239	I	LL	NA	Positive	Negative	5.913437321	N/A
240	I	LL	NA	Negative	Negative	N/A	N/A
241	I	LL	NA	Negative	Negative	N/A	N/A
242	I	LL	NA	Negative	Negative	N/A	N/A
243	I	LL	NA	Negative	Negative	N/A	N/A
244	II	LL	NA	Negative	Negative	N/A	N/A
245	II	LL	NA	Negative	Negative	N/A	N/A
246	II	LL	NA	Negative	Negative	N/A	N/A
247	II	LL	NA	Positive	Positive	6.63902754	6.247222365
248	II	LL	NA	Negative	Negative	N/A	N/A
249	II	LL	NA	Negative	Negative	N/A	N/A
250	II	LL	NA	Negative	Negative	N/A	N/A
251	II	LL	NA	Negative	Negative	N/A	N/A
252	II	LL	NA	Positive	Positive	6.656598956	5.981766166
253	II	LL	NA	Negative	Negative	N/A	N/A
254	II	LL	NA	Positive	Negative	6.571925971	N/A
255	II	LL	NA	Negative	Negative	N/A	N/A
256	II	LL	NA	Negative	Negative	N/A	N/A
257	II	LL	NA	Negative	Negative	N/A	N/A
258	II	LL	NA	Negative	Negative	N/A	N/A
259	II	LL	NA	Positive	Positive	7.045671833	5.672781079
260	II	LL	NA	Negative	Negative	N/A	N/A
261	II	LL	NA	Negative	Negative	N/A	N/A
262	II	LL	NA	Positive	Positive	6.853457123	5.534064158
263	II	LL	NA	Negative	Negative	N/A	N/A
264	II	LL	NA	Negative	Negative	N/A	N/A
265	II	LL	NA	Negative	Negative	N/A	N/A
266	II	LL	NA	Negative	Negative	N/A	N/A
267	II	LL	NA	Negative	Negative	N/A	N/A
268	II	LL	NA	Negative	Negative	N/A	N/A
269	II	LL	NA	Positive	Positive	6.871666739	5.578937081
270	II	LL	NA	Positive	Positive	6.077577102	5.61485802
271	II	LL	NA	Negative	Negative	N/A	N/A
272	II	LL	NA	Negative	Negative	N/A	N/A
273	II	LL	NA	Negative	Negative	N/A	N/A
274	II	LL	NA	Negative	Negative	N/A	N/A

275	II	LL	NA	Negative	Negative	N/A	N/A
276	II	LL	NA	Negative	Negative	N/A	N/A
277	II	LL	NA	Negative	Negative	N/A	N/A
278	II	LL	NA	Negative	Negative	N/A	N/A
279	II	LL	NA	Negative	Negative	N/A	N/A
280	II	LL	NA	Negative	Negative	N/A	N/A
281	II	LL	NA	Negative	Negative	N/A	N/A
282	II	LL	NA	Negative	Negative	N/A	N/A
283	II	LL	NA	Positive	Negative	5.229538269	N/A
284	II	LL	NA	Positive	Positive	7.382233886	6.034529692
285	II	LL	NA	Negative	Negative	N/A	N/A
286	II	LL	NA	Positive	Positive	6.841573453	5.627915422
287	II	LL	NA	Negative	Negative	N/A	N/A
288	II	LL	NA	Negative	Negative	N/A	N/A
289	II	LL	NA	Negative	Negative	N/A	N/A
290	II	LL	NA	Negative	Negative	N/A	N/A
291	II	LL	NA	Negative	Negative	N/A	N/A
292	II	LL	NA	Negative	Negative	N/A	N/A
293	II	LL	NA	Negative	Negative	N/A	N/A
294	I	NL	NA	Negative	Negative	N/A	N/A
295	I	NL	NA	Negative	Negative	N/A	N/A
296	I	NL	NA	Negative	Negative	N/A	N/A
297	I	NL	NA	Negative	Negative	N/A	N/A
298	I	NL	NA	Negative	Negative	N/A	N/A
299	I	NL	NA	Negative	Negative	N/A	N/A
300	I	NL	NA	Negative	Negative	N/A	N/A
301	I	NL	NA	Negative	Negative	N/A	N/A
302	I	NL	NA	Positive	Positive	6.467178011	5.8128259
303	I	NL	NA	Negative	Negative	N/A	N/A
304	I	NL	NA	Negative	Negative	N/A	N/A
305	I	NL	NA	Positive	Negative	6.174722969	N/A
306	I	NL	NA	Negative	Negative	N/A	N/A
307	I	NL	NA	Negative	Negative	N/A	N/A
308	I	NL	NA	Positive	Positive	6.489061105	3.182018156
309	I	NL	NA	Negative	Negative	N/A	N/A
310	I	NL	NA	Negative	Negative	N/A	N/A
311	I	NL	NA	Negative	Negative	N/A	N/A
312	I	NL	NA	Negative	Negative	N/A	N/A
313	I	NL	NA	Negative	Negative	N/A	N/A
314	I	NL	NA	Negative	Negative	N/A	N/A

315	I	NL	NA	Negative	Negative	N/A	N/A
316	I	NL	NA	Negative	Negative	N/A	N/A
317	I	NL	NA	Negative	Negative	N/A	N/A
318	I	NL	NA	Negative	Negative	N/A	N/A
319	I	NL	NA	Negative	Negative	N/A	N/A
320	I	NL	NA	Negative	Negative	N/A	N/A
321	I	NL	NA	Negative	Negative	N/A	N/A
322	I	NL	NA	Negative	Negative	N/A	N/A
323	I	NL	NA	Negative	Negative	N/A	N/A
324	I	NL	NA	Negative	Negative	N/A	N/A
325	I	NL	NA	Negative	Negative	N/A	N/A
326	I	NL	NA	Negative	Negative	N/A	N/A
327	I	NL	NA	Negative	Negative	N/A	N/A
328	I	NL	NA	Negative	Negative	N/A	N/A
329	I	NL	NA	Negative	Negative	N/A	N/A
330	I	NL	NA	Negative	Negative	N/A	N/A
331	I	NL	NA	Negative	Negative	N/A	N/A
332	I	NL	NA	Negative	Negative	N/A	N/A
333	I	NL	NA	Positive	Positive	5.977900429	4.691183835
334	I	NL	NA	Negative	Negative	N/A	N/A
335	I	NL	NA	Negative	Negative	N/A	N/A
336	I	NL	NA	Negative	Negative	N/A	N/A
337	I	NL	NA	Positive	Positive	6.690162346	4.047050062
338	II	NL	NA	Negative	Negative	N/A	N/A
339	II	NL	NA	Negative	Negative	N/A	N/A
340	II	NL	NA	Negative	Negative	N/A	N/A
341	II	NL	NA	Negative	Negative	N/A	N/A
342	II	NL	NA	Negative	Negative	N/A	N/A
343	II	NL	NA	Positive	Negative	5.683305347	N/A
344	II	NL	NA	Positive	Positive	6.677844315	5.98739521
345	II	NL	NA	Negative	Negative	N/A	N/A
346	II	NL	NA	Negative	Negative	N/A	N/A
347	II	NL	NA	Negative	Negative	N/A	N/A
348	II	NL	NA	Negative	Negative	N/A	N/A
349	II	NL	NA	Negative	Negative	N/A	N/A
350	II	NL	NA	Negative	Negative	N/A	N/A
351	II	NL	NA	Positive	Positive	7.06187158	5.981174839
352	II	NL	NA	Negative	Negative	N/A	N/A
353	II	NL	NA	Negative	Negative	N/A	N/A
354	II	NL	NA	Negative	Negative	N/A	N/A

355	II	NL	NA	Negative	Negative	N/A	N/A
356	II	NL	NA	Negative	Negative	N/A	N/A
357	II	NL	NA	Negative	Negative	N/A	N/A
358	II	NL	NA	Negative	Negative	N/A	N/A
359	II	NL	NA	Negative	Negative	N/A	N/A
360	II	NL	NA	Negative	Negative	N/A	N/A
361	II	NL	NA	Negative	Negative	N/A	N/A
362	II	NL	NA	Negative	Negative	N/A	N/A
363	II	NL	NA	Negative	Negative	N/A	N/A
364	II	NL	NA	Negative	Negative	N/A	N/A
365	II	NL	NA	Negative	Negative	N/A	N/A
366	II	NL	NA	Negative	Negative	N/A	N/A
367	II	NL	NA	Negative	Negative	N/A	N/A
368	II	NL	NA	Negative	Negative	N/A	N/A
369	II	NL	NA	Negative	Negative	N/A	N/A
370	II	NL	NA	Negative	Negative	N/A	N/A
371	II	NL	NA	Negative	Negative	N/A	N/A
372	II	NL	NA	Negative	Negative	N/A	N/A
373	II	NL	NA	Negative	Negative	N/A	N/A
374	II	NL	NA	Negative	Negative	N/A	N/A
375	II	NL	NA	Negative	Negative	N/A	N/A
376	II	NL	NA	Negative	Negative	N/A	N/A
377	II	NL	NA	Negative	Negative	N/A	N/A
378	II	NL	NA	Negative	Negative	N/A	N/A
379	II	NL	NA	Negative	Negative	N/A	N/A
380	II	NL	NA	Negative	Negative	N/A	N/A
381	II	NL	NA	Negative	Negative	N/A	N/A
382	II	NL	NA	Negative	Negative	N/A	N/A
383	II	NL	NA	Negative	Negative	N/A	N/A
384	II	NL	NA	Negative	Negative	N/A	N/A
385	II	NL	NA	Positive	Positive	7.288379531	5.770325207
386	II	NL	NA	Negative	Negative	N/A	N/A

Table S2. The primers used for gene expression real-time PCR

Gene ID	Primer Name	Primer sequence	References
LOC5577953	Spaetzle1A Forward	5'-GACAAAGAACGGATGCCAAT-3'	1
	Spaetzle1A Reverse	5'-TTCGAGGGAATCATTTGGAC-3'	
LOC5578273	Toll 1B Forward	5'-TCTCATCAGGATTCCACAAC-3'	2
	Toll 1B Reverse	5'-CGGTTGAAATTCCGACGAAG-3'	
LOC5569526	Rel 1A Forward	5'-TGGTGGTGGTGTCCTGCGTAAC-3'	3
	Rel 1A Reverse	5'-CTGCCTGGCGTGACCGTATCC-3'	
LOC5565922	Cactus Forward	5'-AGACAGCCGCACCTTCGATTCC-3'	1
	Cactus Reverse	5'-CGCTTCGGTAGCCTCGTGGATC-3'	
LOC5576380	Domeless Foward	5'-AAACGGTGGCAAAATGAACT-3'	4
	Domeless Reverse	5'-CATACAGCCGGCTTTCTTCT-3'	
LOC5576502	Hopscotch Foward	5'-ACAGGCACAGGCCGAAAA-3'	5
	Hopscotch Reverse	5'-CCGTTGGACAGCTCGATAAAG-3'	
LOC5579515	PIAS 2 Foward	5'-GCTGCAACGCATGAAAACTA-3'	4
	PIAS 2 Reverse	5'-CAGACGGGACAGTTCCAAGT-3'	
LOC5578608	Caspar Forward	5'-GAATCCGAGCGAGCCGATGC-3'	1
	Caspar Reverse	5'-CGTAGTCCAGCGTTGTGAGGTC-3'	
LOC5569427	Rel 2 Forward	5'-TTTGAATGTGCTGTTGGGTC-3'	6
	Rel 2 Reverse	5'-GAATGTTGTTTCCGTGCTTA-3'	
LOC5572865	Imd Forward	5'-ATCCCGACATCTGGGATATG-3'	3
	Imd Reverse	5'-GGGTTGACTTTGTCGTCGTT-3'	
LOC5576901	PGRP-LC Forward	5'-CACACACCGCAACAGAAAAC-3'	7
	PGRP-LC Reserse	5'-AACGTAAGCGTTTCCGTCAC-3'	
LOC5579094	Defensin C Forward	5'-TGTTCGCTCTTACGCCAACT-3'	1
	Defensin C Reverse	5'-ATCTCCTACACCGAACCCACT-3'	

LOC5564826	Cecropin D Forward	5'-ATGAACTTCACTAAGCTGTT-3'	3
	Cecropin D Reverse	5'-TCATTTTCCAATCGCTTTTAT-3'	
LOC110676293	Lysozyme Forward	5'-CCACGGCAACTGGATATGTCT-3'	8
	Lysozyme Reverse	5'-TCTGCGTCACCTTGGTGGTAT-3'	
LOC5572090	S7 Forward	5’-GGGACAAATCGGCCAGGCTATC-3’	1
	S7 Reverse	5’-TCGTGGACGCTTCTGCTTGTTG-3’	

SI References

1. Xi, Z., Ramirez, J. L. & Dimopoulos, G. The *Aedes aegypti* toll pathway controls dengue virus infection. PLoS Pathogens 4, e1000098 (2008).
2. Shin, S. W., Bian, G. & Raikhel, A. S. A toll receptor and a cytokine, Toll5A and Spz1C, are involved in toll antifungal immune signaling in the mosquito *Aedes aegypti*. Journal of Biological Chemistry 281, 39388-39395 (2006).
3. Pan, X. et al. *Wolbachia* induces reactive oxygen species (ROS)-dependent activation of the Toll pathway to control dengue virus in the mosquito *Aedes aegypti*. Proceedings of the National Academy of Sciences 109, E23-E31 (2012).
4. Souza-Neto, J. A., Sim, S. & Dimopoulos, G. An evolutionary conserved function of the JAK-STAT pathway in anti-dengue defense. Proceedings of the National Academy of Sciences 106, 17841-17846 (2009).
5. Carvalho-Leandro, D. et al. Immune transcript variations among *Aedes aegypti* populations with distinct susceptibility to dengue virus serotype 2. Acta Tropica 124, 113-119 (2012).
6. Wang, Y.-H. et al. A critical role for CLSP2 in the modulation of antifungal immune response in mosquitoes. PLoS Pathogens 11, e1004931 (2015).
7. Ramirez, J. L., Muturi, E. J., Barletta, A. B. & Rooney, A. P. The *Aedes aegypti* IMD pathway is a critical component of the mosquito antifungal immune response. Developmental & Comparative Immunology **95**, 1-9 (2019).
8. Ramirez, J. L. *et al.* Reciprocal tripartite interactions between the *Aedes aegypti* midgut microbiota, innate immune system and dengue virus influences vector competence. PLoS neglected tropical diseases **6**, e1561 (2012).

Table S3. Relative fold changes in immune gene expression of *Ae aegypti*. LL: low larval nutrition, NL: normal larval nutrition, LA: low adult nutrition, NA: normal adult nutrition, Spa: Spaetzle 1A, Tol: Toll 1B, Rel1: Rel 1A, Cac: Cactus, Dom: Domeless, Hop: Hopscotch, PIA: PIAS 2, Cas: Caspar, Rel2: Rel 2, PGRP: PGRP-LC, Def: Defensin C, Cec: Cecropin D, Lyso: Lysozyme. Data were rounded to 2 decimal places.

ID	Larval nutrition	Adult nutrition	Infection status	Spa	Tol	Rel1	Cac	Dom	Hop	PIA	Cas	Rel2	Imd	PGRP	Def	Cec	Lyso
1	LL	LA	positive	0.19	0.76	0.42	2.11	0.54	0.52	0.97	0.81	0.63	0.46	0.75	0.77	3.82	0.19
2	LL	LA	positive	0.35	0.40	0.52	2.96	1.40	0.28	2.11	1.28	0.56	0.32	0.41	0.80	0.63	0.23
3	LL	LA	positive	0.18	0.13	0.37	1.83	0.57	0.20	0.68	0.52	0.34	0.20	0.96	0.47	0.27	0.52
4	LL	LA	negative	0.18	0.16	0.27	1.96	1.11	0.50	1.10	0.84	0.33	0.22	0.70	0.48	0.10	0.10
5	LL	LA	negative	3.38	5.29	3.83	0.87	1.35	0.83	2.04	1.19	2.67	0.54	2.43	0.90	1.02	1.98
6	LL	LA	negative	0.12	0.23	0.48	1.16	0.60	0.32	0.97	0.45	0.61	0.22	0.22	0.19	0.24	0.54
7	NL	LA	positive	0.67	0.36	0.60	0.77	1.10	1.11	0.96	0.57	2.18	1.49	0.72	15.57	28.73	0.60
8	NL	LA	positive	1.53	0.30	0.40	0.86	1.12	0.46	0.68	0.40	0.73	0.96	1.77	1.02	0.63	0.53
9	NL	LA	positive	1.54	0.79	0.59	0.76	0.85	0.55	0.59	0.42	0.94	0.65	0.66	0.86	4.28	0.66
10	NL	LA	negative	2.55	4.15	0.66	0.80	1.05	1.35	0.67	0.85	2.67	1.13	1.45	2.94	11.61	0.85
11	NL	LA	negative	2.81	0.96	1.00	1.12	1.08	1.70	1.30	0.72	0.92	1.00	2.36	0.93	0.53	1.16
12	NL	LA	negative	23.55	31.63	1.40	0.24	2.41	4.06	0.36	0.41	10.37	3.21	4.02	5.99	7.84	2.29
13	LL	NA	positive	1.75	0.23	0.27	1.11	0.63	0.39	1.00	1.62	0.56	0.73	0.60	2.01	0.32	1.81
14	LL	NA	positive	2.31	0.74	0.20	0.72	0.50	0.20	0.71	1.04	0.32	0.24	0.19	0.75	0.44	1.04
15	LL	NA	positive	2.14	1.26	0.24	0.37	0.20	0.07	0.29	0.22	0.92	0.62	0.69	1.01	1.50	0.94
16	LL	NA	negative	0.65	0.13	0.21	0.63	0.83	0.22	0.47	0.52	0.45	0.81	0.43	0.73	0.04	0.16
17	LL	NA	negative	2.21	0.28	0.42	1.07	0.58	0.36	0.79	1.75	0.86	0.75	0.84	1.79	0.22	0.40
18	LL	NA	negative	0.91	0.38	0.17	0.59	0.38	0.27	0.31	0.54	0.75	0.53	0.50	1.24	0.22	1.27
19	NL	NA	positive	3.43	16.58	0.33	0.08	0.26	0.33	0.17	0.22	1.92	1.37	0.47	1.88	2.11	0.04
20	NL	NA	positive	5.53	25.90	0.78	0.40	0.80	0.32	0.63	0.58	3.09	1.48	1.97	0.78	2.72	7.39
21	NL	NA	positive	5.15	3.38	0.49	0.90	0.58	0.31	0.77	0.60	2.06	0.68	0.35	0.43	0.36	1.70
22	NL	NA	negative	0.22	0.48	0.71	0.76	2.41	0.48	0.70	1.29	2.32	2.47	1.06	0.30	0.02	4.14

23	NL	NA	negative	21.64	200.81	2.70	0.24	1.63	3.17	0.63	0.27	10.45	2.92	1.41	66.60	629.91	3.46
24	NL	NA	negative	4.10	59.54	1.27	1.82	1.06	1.05	1.38	0.46	5.21	4.03	1.27	7.66	14.74	6.61

Table S4. List of models used in statistical analyses. GLM: generalized linear model, CPHM: Cox-proportional hazards model, ANCOVA: analysis of covariance, LM: linear model, N/A: not applicable, AIC: Akaike information criterion, AICc: corrected AIC for small sample size. A global model shows all the predictors included in the initial model. Model selection was performed based on the lowest value of AIC or AICc. A best model shows the best fitted model after model selection.

Model	Response	Predictor	Model selection
Gaussian GLM	wing length	block + larval nutrition + adult nutrition + larval nutrition x adult nutrition	N/A
Global hurdle model	fecundity	block + larval nutrition + adult nutrition + wing length + blood feeding (infectious/non-infectious blood meal) + larval nutrition x adult nutrition x blood-feeding + larval nutrition x adult nutrition + larval nutrition x blood feeding + adult nutrition x blood feeding + larval nutrition x wing length	AIC
Best hurdle model	fecundity	block + larval nutrition + adult nutrition + wing length + blood feeding + larval nutrition x adult nutrition x blood feeding + larval nutrition x adult nutrition + larval nutrition x blood feeding + adult nutrition x blood feeding	AIC
Global CPHM	survival	block + larval nutrition + adult nutrition + size group + blood feeding + larval nutrition x adult nutrition x blood feeding + larval nutrition x adult nutrition + larval nutrition x blood feeding + adult nutrition x blood feeding + larval nutrition x size group	AIC
Best CPHM	survival	block + larval nutrition + adult nutrition + blood feeding+ larval nutrition x adult nutrition x blood feeding + larval nutrition x adult nutrition + larval nutrition x blood feeding + adult nutrition x blood feeding	AIC
Survival curves	survival	larval nutrition or adult nutrition or blood feeding	N/A
ANCOVA: LM with covariate	fecundity	Wing length + larval nutrition or adult nutrition or blood feeding + wing length x larval nutrition or adult nutrition or blood feeding	N/A
Global binomial GLM	dengue viral status (positive/negative) in body or leg	block + larval nutrition + adult nutrition + wing length + survival + larval nutrition x adult nutrition + larval nutrition x wing length + adult nutrition x survival	AIC
Best binomial GLM	dengue viral status (positive/negative) in body or leg	for body: adult nutrition + wing length; for leg: adult nutrition + wing length + survival + adult nutrition x survival	AIC
Global gaussian GLM	dengue viral titer in body or leg	block + larval nutrition + adult nutrition + wing length + survival + larval nutrition x adult nutrition + larval nutrition x wing length + adult nutrition x survival	AICc
Best gaussian GLM	dengue viral titer in body or leg	for body: block + larval nutrition + adult nutrition + survival + adult nutrition x survival; for leg: block + adult nutrition + survival + adult nutrition x survival	AICc

Global gaussian GLM	relative fold change in immune gene expression	larval nutrition + adult nutrition + dengue infection status (positive or negative) + larval nutrition x adult nutrition + larval nutrition x infection status + adult nutrition x infection status	AICc
Best gaussian GLM	relative fold change in immune gene expression	Spaetzle ~ larval nutrition; Toll1 ~ larval nutrition; Rel1 ~ infection status; Cactus ~ larval nutrition + adult nutrition + larval nutrition x adult nutrition; Domeless ~ larval nutrition + infection status + larval nutrition x infection status; Hopscotch ~ larval nutrition + infection status + larval nutrition x infection status; PIAS2 ~ adult nutrition; Caspar ~ larval nutrition; Rel2 ~ larval nutrition + infection status + larval nutrition x infection status; Imd ~ larval nutrition + infection status + adult nutrition + larval nutrition x infection status; PGRP-LC ~ larval nutrition+ infection status + adult nutrition; Defensin ~ larval nutrition; Cecropin ~ larval nutrition; Lysozyme ~ larval nutrition + adult nutrition + larval nutrition x adult nutrition	AICc
