# Supplementary material for: Nutritional stress compromises mosquito fitness and antiviral immunity, while enhancing dengue virus infection susceptibility
Source: Commun Biol. 2023 Nov 6;6:1123. doi: 10.1038/s42003-023-05516-4 (PMC10628303; doi:10.1038/s42003-023-05516-4)
Supplement: Supplementary file 3 — Description of Additional Supplementary Files [file 42003_2023_5516_MOESM3_ESM.pdf]

Description of Additional Supplementary Files

File name: Supplementary Data 1

Description: Life history traits of *Aedes aegypti*. LL: low larval nutrition, NL: normal larval nutrition, LA: low adult nutrition, NB: non-infectious blood meal, IB: dengue infectious blood meal, NA: normal adult nutrition, N/A: not available, dpbf: day post blood feeding.
