# Supplementary material for: Nutritional stress compromises mosquito fitness and antiviral immunity, while enhancing dengue virus infection susceptibility
Source: Commun Biol. 2023 Nov 6;6:1123. doi: 10.1038/s42003-023-05516-4 (PMC10628303; doi:10.1038/s42003-023-05516-4)
Supplement: Supplementary file 1 — Peer Review File [file 42003_2023_5516_MOESM1_ESM.pdf]

nature portfolio

Peer Review File

Nutritional stress compromises mosquito fitness and antiviral immunity, while enhancing dengue virus infection susceptibility

Open Access This file is licensed under a Creative Commons Attribution 4.0 International License, which permits use, sharing, adaptation, distribution and reproduction in any medium or format, as long as you give appropriate credit to the original author(s) and the source, provide a link to the Creative Commons license, and indicate if changes were made. In the cases where the authors are anonymous, such as is the case for the reports of anonymous peer reviewers, author attribution should be to 'Anonymous Referee' followed by a clear attribution to the source work. The images or other third party material in this file are included in the article's Creative Commons license, unless indicated otherwise in a credit line to the material. If material is not included in the article's Creative Commons license and your intended use is not permitted by statutory regulation or exceeds the permitted use, you will need to obtain permission directly from the copyright holder. To view a copy of this license, visit http://creativecommons.org/licenses/by/4.0/.

Reviewers' comments:

Reviewer #1 (Remarks to the Author):

In this experimentally impressive study, the authors assess how mosquito nutrition during the larval and adult life stages impacts life history and the ability to sustain a dengue infection. From an experimental perspective, this is an ambitious project that goes beyond prior studies that have assessed either larval or adult nutrition, and instead goes on to uncover the interaction between the two. Overall, the authors conclude that larval and adult nutritional stress negatively affects body size and survival, and interacts to reduce fecundity. Moreover, nutritional stress as an adult, but not as larvae, renders mosquitoes more susceptible to an infection with the dengue virus.

Although the study is experimentally ambitious and the findings of significant importance – and for that reason I am supportive of the manuscript – the manuscript suffers from a significant messaging issue that will reduce its impact. In other words, the manuscript is difficult to read and gets bogged down in the statistical details to the point that the true message is difficult to discern. Moreover, there are several seeming contradictions that are unexplained, and the magnitude of many of the differences are not stated (just because something is statistically significant does not mean it is biologically significant).

1. On the messaging: the entire results section is about the statistics. Significant effects and significant interactions are clearly noted, but how they relate to the biology is not mentioned or prioritized. Some of this is tucked in the discussion. The discussion is meant to be a place where the findings are analyzed in the context of the published literature. However, a good number of the paragraphs in the discussion lack references, which means that they are interpreting the results instead of discussing the findings relative what is already known. Many of these sentences should be weaved into the results so that the reader understands what, for example, a given interaction signifies.

2. Seemingly contradicting statements: the manuscript contains some seemingly contradicting statements that may result from the emphasis in the statistics instead of the biology. Here are some examples:
2a. Lines 236-243 – "positive effect of the interaction between larval and adult nutritional stress on fecundity" seemingly contradicts with "presence/absence of eggs was negatively affected by adult nutritional stress". So, if a mosquito lays eggs, it will lay more eggs if nutritionally stressed but when nutritionally stressed it is more likely to not lay eggs?
2b. Lines 243-247 – "neither larval and adult nutritional stress nor ingestion of an infectious bloodmeal independently increased death risk of mosquitoes" seemingly contradicts with "nutritional stress significantly shortened adult survival as shown in survival curves". So, nutritional stress does not increase the likelihood of dying but decreases longevity?
2c. Mosquitoes with larger wings emerge from larvae with normal food (table 1), yet mosquitoes with larger wings are more fecund while mosquitoes that receive low nutrition throughout (LL X LA) are more fecund as well (figure 2A). This seems contradictory.

3. The tables would be more useful if they showed the values for the specific combinations. As written, NL combines the values from NA and LA, etc. So, for example, the reader cannot see the difference between NL X NA and NL X LA.

4. The statistical models used are not clear. The authors should include an appendix with the models so that the reader can understand the comparisons being made. As written, the comparisons for interaction effects are not clear, or what factors are accounted for.

5. Conceptually, there are several theories the authors should consider. In terms of fecundity, given the findings on low nutrition the authors should consider the terminal investment theory. As for the

carry over effects associated with metamorphosis that are mentioned in the introduction, the authors should consider the adaptive decoupling theory.

6. I recommend that the authors refrain, or more carefully claim that this is the first time something is done. Yes, this is likely the first time this combination is done (and impressively done) but many of the parameters have already been measured individually. The major benefit of this study is uncovering the interactions.

7. In the introduction, I recommend that the authors avoid the “but see” parentheticals. It is unclear what there is to see. If it is important, then it should be stated.

Reviewer #2 (Remarks to the Author):

Manuscript: COMMSBIO-22-1863-T

The authors studied here how nutritional stress (on larval and adult stages) would impact fitness and vector competence of mosquitoes towards dengue virus. It seems that it is just a revisit of several previous studies in the field. Having said that, the manuscript is well written, and the statistical analyses are robust but there are important points that need careful considerations to promote adequate conclusions.

Major comments

Negative points include the use of F19th generation of mosquitoes from a laboratory. Getting far away from field conditions removes real world responses and make findings quite artificial. The same relates to this particular DENV4 isolate. Although it is a reference virus, it might not resemble what mosquitoes would find in a real world.

It is not clear whether DENV4 titers were related to a mosquito gene (RPS for instance). Diets (as showed here) would manipulate mosquito size and therefore, infection rates should be relative, to correct this bias. Data presented in Table 2 do not show this.

Reviewer #3 (Remarks to the Author):

The paper entitled “Nutritional stress compromises mosquito fitness and antiviral immunity but enhances dengue virus infection susceptibility" by Dr Yan et al use clear figures to show a comprehensive and interesting study about the impact of nutrition provision at different life stages of mosquitoes on their life history, immunity and viral infection, whereas these three aspects and their results lack novelty. The authors also provided studies about their interactions which are interesting and have some novelty, like “does low nutrition in the larval stage can be rescued in the later adult stage?” but the manuscript doesn’t use enough words to emphasize this part. At least, from the Abstract people couldn’t grab the significance of this study and its novelty. Besides, the language of this manuscript should be improved. Some sentences are very confusing. The authors should be honest/careful when they claim “first” about their study (line 220, 275), as there are many nutrition studies about mosquitoes in different aspects and the authors also recognize this and have cited a lot of them. The result part is very tedious and difficult for people to read and understand, especially the immune gene expression part, adding one or two sentences about their main result will be much better. The discussion on the other hand, contains a lot of results but lacks information about the significance and implication of these results. For example, how does nutrition provision affect

mosquito distribution and viral transmissibility? Besides, the methods are unclear about how many mosquitoes and replicates were tested in the study, did the authors have separate replicates about viral titers? How did they do non-infectious blood feeding? Did they use mock media? In general, the authors should put more effort into improving this manuscript.
Some small issues:
Line 39: how can adult nutritional stress affect adult body size?
Line 133: what is the unit of female fecundity?

Reviewer #4 (Remarks to the Author):

The role of variation in relevant environmental factors on vector-borne disease transmission and the underlying mechanisms is an active field of investigation. Questions along this line are important as the majority of work done to date has focused on characterizing mosquito-pathogen interactions and mosquito immunity under highly controlled, laboratory conditions that are not reflective of variation mosquitoes may experience in the field. This study focuses on the effect of a biotic factor, such as food availability, in the larval and adult environment, and how variation in this factor affects mosquito fitness (survival and reproduction), immunity, and dengue vector competence. Strengths of the study include investigating the interactive effect of larval and adult nutrition, as well as infection status, on mosquito fitness and life history. Overall, the authors found that larval and adult nutrition and infection status had impact on these response variables. Weaknesses include potential lack of biological replication, and how the authors executed the arbovirus infection and measured the immune response, which will require more in-depth discussion than what is currently presented. Thus, we have several major concerns that need to be addressed before this manuscript can be considered for publication in this journal.

Major Concerns:

• Justification for the sugar treatments being different- adults have access to sugar of differing concentrations, but access was ad libitum. How do we know that they consumed differing amount of sugar if compensatory feeding is occurring?

• Why only one bloodmeal? Especially since it is not clear how much aegypti rely on sugar sources in the field.

• We have substantial concerns about when immunity was measured with respect to infection and the lack of characterizing dynamics (of both the infection and the immune response). First, it was really unclear from the introduction and methods why gene expression 10 days after the challenge would be representative of the level of elicitation of immune signaling pathways during the infection process. Mosquito immune responses to infection are initiated 24-48 hr post-infection, depending on the pathogen, and involve midgut and systemic responses. It is unclear what characterizing the immune response at 10 days tells us about the response to infection. Further, characterizing infection phenotypes from mosquitoes that die naturally means we have a mixture of infection stages (midgut, disseminated, salivary glands) in the final sample leading to a lot of variation probably in the proportion of mosquitoes infected or with disseminated infections and the final titer. With this design, there is a loss of mechanistic insight into where the bottle necks in infection are occurring and how the treatments affected the nature of these bottlenecks. These experimental design issues likely result in the disconnect between the effects of adult nutrition on dengue infection phenotypes with the effects of the larval environment on arbovirus immunity. In end, we still do not have a resolved picture of how the effects of these treatments connect across immunity and arbovirus susceptibility.

• The results section needs to rewritten to be explained in terms of the biological hypotheses instead of description of the model outputs. In particular, the interactions needs to be described. For example there is a three way interaction between LL, LA, and IB affecting fecundity (Line 139-140). What does that mean exactly? The effect of larval diet on fecundity depends on the nutrition the mosquito

receives as an adult and whether the blood meal is infected or not...but how. Is it compounding? The results also are highly repetitive with the data that are presented in the figures and tables. The treatment means should appear in on or the other not both.

• Replication? Not clear this was done rigorously. The only time replication is mentioned is in the immune assay section of the methods. For full biological replication to occur, the authors would have needed to replicate all experimental treatments (the larval portion of the experiment, the adult portion of the experiment, and the infections with different generations of mosquitoes). Thus, I suspect the replication they are referring to is actually pseudoreplication, which is a problem. Also, a clearer explanation of numbers of groups and sample sizes within each treatment and component of the study (life history, arbovirus infection, and immunity)

• The comparisons across treatments could also be clearer – especially in the adult stage, which consists of adults that receive high food but were from either a H / L food larval stage vs. adults that receive low food but were from either a H / L food larval stage. Sometimes adults are averaged across the larval stage…is this because the larval manipulation was not significant? If so, it would be helpful to state the non-significant effects as well as the significant effects. It would also be nice to know what your model structure is for each model (the factors included in the model were given, but you did not specify what interactions were explored or the models that were compared using AIC. In the supplement, it would be great to include this information. Also the summary tables are averaged across the main treatments and it would be nice to have all of the treatments broken out when presenting raw data means.

• Finally, why not consider effects of the extrinsic incubation period? They open in the introduction by framing their study in terms of vectorial capacity. Vector competence has a fairly weak effect on overall transmission potential when compared to other variables in vectorial capacity, like EIP, when sensitivity analyses have been conducted. Variation in adult reserves and carry over effects on these reserves could also have big impacts on this response variable (e.g., see: https://parasitesandvectors.biomedcentral.com/articles/10.1186/s13071-014-0593-4 and https://royalsocietypublishing.org/doi/full/10.1098/rspb.2016.0298).

Minor Concerns:
• Introduction could be organized a bit differently. Focus second paragraph only on life history traits and third paragraph only on pathogen traits. Right now, the second paragraph has both. Also in the third paragraph, there is no description of EIP…which is significantly more important than vector competence.

• Line 29- There has been previous work investigating the effect of larval nutrition on immunity in this species including study cited [29] and at least one looking at adult nutrition: https://journals.plos.org/plosntds/article?id=10.1371/journal.pntd.0005158. What we know from these studies and how this work differs needs to be more clearly spelled out in the introduction.

• Line 63- First sentence is vague-

• Line 89- if only a small proportion of mosquitoes are infected to we expect infection associated decreases in fecundity to really shape density?

• Only one blood meal and associated egg lay were measured- how do they think this affects their survival and fecundity measures?

• Figs 2, 5ab- again not clear what interactions really look like. Plotting data would be more helpful.

• Fig 3- it would be helpful to see all 8 treatments on a single plot or the 4 treatments broken up by larval nutrition.

• Table 3- Not clear what this is a table of: are these the final model for each gene? So a "-" means that a parameter was not in the final model and the NS is that it was in the final model but not significant?

• Should cite: https://www.frontiersin.org/articles/10.3389/fmicb.2022.886787/full around line 234

• Does rpS7 change with experimental treatment? If so, this is not a good housekeeping gene (see https://royalsocietypublishing.org/doi/full/10.1098/rspb.2012.0638?rss=1). It also is unclear how the authors normalized expression of target genes to this gene?

Things to add to the discussion:

• Why does low adult food impact the probability of laying eggs only? I would expect low teneral reserves to interact with low adult food to impact this response variable. There are multiple studies that have demonstrated that mosquitoes of low teneral reserves might need multiple bloodmeals to produce a batch of eggs (see https://pubmed.ncbi.nlm.nih.gov/22300806/). Also, how do you know all females were successfully mated?

• No effect of larval nutrition on vector competence…there has been a lot of work on this previously, showing that this should have an effect (Barry Alto's work). Why not here? Sugar provided ad libitum?

• Larval nutrition affects arbovirus immunity but adult nutrition affects DENV vector competence phenotypes…why? This mismatch and when the immune system was measured makes it hard to definitely say immunosuppression resulted in the reduced vector competence observed. Need to build in justification for why 10 days is a relevant time point to measure and what this time point is capturing in your study (e.g., end point immunity)?

• Need a limitations paragraph in the discussion section where you reflect on the limits of your study and implications for your results. Every study has limitations…these should be transparently addressed.

Response to Reviewer #1

Reviewer #1 (Remarks to the Author):

In this experimentally impressive study, the authors assess how mosquito nutrition during the larval and adult life stages impacts life history and the ability to sustain a dengue infection. From an experimental perspective, this is an ambitious project that goes beyond prior studies that have assessed either larval or adult nutrition, and instead goes on to uncover the interaction between the two. Overall, the authors conclude that larval and adult nutritional stress negatively affects body size and survival, and interacts to reduce fecundity. Moreover, nutritional stress as an adult, but not as larvae, renders mosquitoes more susceptible to an infection with the dengue virus.

Although the study is experimentally ambitious and the findings of significant importance – and for that reason I am supportive of the manuscript – the manuscript suffers from a significant messaging issue that will reduce its impact. In other words, the manuscript is difficult to read and gets bogged down in the statistical details to the point that the true message is difficult to discern. Moreover, there are several seeming contradictions that are unexplained, and the magnitude of many of the differences are not stated (just because something is statistically significant does not mean it is biologically significant).

Response: Thank you very much for the appreciation and comments, especially the constructive criticism on messaging issues. We agree with the reviewer that improving the messaging would enhance the impact of this manuscript, and in light of these comments, we have intensively modified and improved our manuscript. Specific modifications /improvements can be seen below.

1. On the messaging: the entire results section is about the statistics. Significant effects and significant interactions are clearly noted, but how they relate to the biology is not mentioned or prioritized. Some of this is tucked in the discussion. The discussion is meant to be a place where the findings are analyzed in the context of the published literature. However, a good number of the

paragraphs in the discussion lack references, which means that they are interpreting the results instead of discussing the findings relative what is already known. Many of these sentences should be weaved into the results so that the reader understands what, for example, a given interaction signifies.

Response: We agree with the reviewer and have thoroughly rewritten the results and discussion sections by following these suggestions.

2. Seemingly contradicting statements: the manuscript contains some seemingly contradicting statements that may result from the emphasis in the statistics instead of the biology. Here are some examples:

2a. Lines 236-243 – “positive effect of the interaction between larval and adult nutritional stress on fecundity” seemingly contradicts with “presence/absence of eggs was negatively affected by adult nutritional stress”. So, if a mosquito lays eggs, it will lay more eggs if nutritionally stressed but when nutritionally stressed it is more likely to not lay eggs?

Response: Thank you for this comment. We have rewritten this section of the discussion extensively to provide more context and clarity. The analysis indeed first looks at egg laying as a binary outcome (did they lay eggs or not, which was affected by adult nutrition), and then of those that did lay eggs, what variables affected the abundance of eggs (which was lower for larval and adult nutritional stress and exposure to an infectious bloodmeal).

2b. Lines 243-247 – “neither larval and adult nutritional stress nor ingestion of an infectious bloodmeal independently increased death risk of mosquitoes” seemingly contradicts with “nutritional stress significantly shortened adult survival as shown in survival curves”. So, nutritional stress does not increase the likelihood of dying but decreases longevity?

Response: Thank you for the careful reading. We first fitted survival curves for larval nutrition, adult nutrition and blood feeding respectively to compare if there is significant difference between two levels of these three treatments. We found while there was no significant difference for larval nutrition (low vs normal) and blood feeding (infectious vs noninfectious blood meal), adult nutrition was significant with mosquito survival being significantly reduced by low adult nutrition. Next, we further explored the impacts of multiple factors and their interactive effects on survival by including

all three treatment terms, wing length and their interactions in Cox Proportional Hazards models and found none of the explanatory terms had a significant effect, but their interactions showed significant effects, probably due to that their interactive effects are much stronger than their single effect and masked their single effects. We have now rewritten this part to clarify this issue (L164-182).

2c. Mosquitoes with larger wings emerge from larvae with normal food (table 1), yet mosquitoes with larger wings are more fecund while mosquitoes that receive low nutrition throughout (LL X LA) are more fecund as well (figure 2A). This seems contradictory.

Response: The interpretation of log rations for interactions is slightly different, where a positive ratio for interaction means the individual (negative) effects are increased (i.e., more negative). We now add a supplementary figure with fecundity values per treatment level to present this in a different way as well (see Fig. S2).

3. The tables would be more useful if they showed the values for the specific combinations. As written, NL combines the values from NA and LA, etc. So, for example, the reader cannot see the difference between NL X NA and NL X LA.

Response: we updated Table 1 and 2 to provide the suggested information.

4. The statistical models used are not clear. The authors should include an appendix with the models so that the reader can understand the comparisons being made. As written, the comparisons for interaction effects are not clear, or what factors are accounted for.

Response: Thank you for the suggestion. We have added a Table S4 to list the statistical models used in this study, specifying which is the response, which are the predictors including what specific interactions were included in each model.

5. Conceptually, there are several theories the authors should consider. In terms of fecundity, given the findings on low nutrition the authors should consider the terminal investment theory. As for the

carry over effects associated with metamorphosis that are mentioned in the introduction, the authors should consider the adaptive decoupling theory.

Response: Thank you for the recommendation. The residual reproductive value (RRV) from terminal investment theory cannot be measured in current setting of our study. Even if low nutrition could roughly represent low RRV, our results did not support this theory, with mosquitoes having low RRV showing lower fecundity. We did address this theory in the context of limitations of the current study in the discussion, and we think a future study that tests this theory in mosquitoes by looking at reproductive investments later in life would be of interest.
The adaptive decoupling theory proposes that metamorphosis is an adaptation for optimizing expression of traits across life stages that experience opposing selection pressures. We do not believe our data allow us to test this concept as such, but we have used this to flesh out the section on carry-over effects further.

6. I recommend that the authors refrain, or more carefully claim that this is the first time something is done. Yes, this is likely the first time this combination is done (and impressively done) but many of the parameters have already been measured individually. The major benefit of this study is uncovering the interactions.

Response: Thank you for the reminder and appreciation. We refrained from using “first”, explained the major benefit, and tuned down our statements (L253-257, 341).

7. In the introduction, I recommend that the authors avoid the “but see” parentheticals. It is unclear what there is to see. If it is important, then it should be stated.

Response: removed.

Response to Reviewer #2

Reviewer #2 (Remarks to the Author):

Manuscript: COMMSBIO-22-1863-T

The authors studied here how nutritional stress (on larval and adult stages) would impact fitness and vector competence of mosquitoes towards dengue virus. It seems that it is just a revisit of several previous studies in the field. Having said that, the manuscript is well written, and the statistical analyses are robust but there are important points that need careful considerations to promote adequate conclusions.

Response: Thank you very much for the comments and appreciation.

Major comments

Negative points include the use of F19th generation of mosquitoes from a laboratory. Getting far away from field conditions removes real world responses and make findings quite artificial. The same relates to this particular DENV4 isolate. Although it is a reference virus, it might not resemble what mosquitoes would find in a real world.

Response: We agree with reviewer that the lab strains of mosquitoes and virus may not resemble real world situations. We acknowledged this limitation in the new paragraph for limitations/assumptions in discussion section (L418-443).

It is not clear whether DENV4 titers were related to a mosquito gene (RPS for instance). Diets (as showed here) would manipulate mosquito size and therefore, infection rates should be relative, to correct this bias. Data presented in Table 2 do not show this.

Response: You are right that diets manipulate mosquito size and so the effect of diets on infection rate/prevalence could co-variate with the effect of mosquito size. We considered this in our binomial models for infection status/prevalence (infected or uninfected as the response) by including nutritional levels as the main predictors, mosquito size and its interaction with larval nutrition as the covariates. Therefore, the effect of mosquito size mediated by larval nutrition on infection rate/prevalence should have been accounted in our models. We found a significant negative effect of mosquito size (wing length) on infection status/prevalence, that is, larger-sized mosquitoes having lower infection prevalence/rate, but no significant effect for larval nutrition nor the interaction between larval nutrition and mosquito size (Fig. 5A &B). Table 2 has been updated to show interactions between nutritional levels. We agree that DENV4 titers may be regulated by some

mosquito genes and would like to investigate this interesting topic in the future.

Reviewer #3 (Remarks to the Author):

The paper entitled “Nutritional stress compromises mosquito fitness and antiviral immunity but enhances dengue virus infection susceptibility" by Dr Yan et al use clear figures to show a comprehensive and interesting study about the impact of nutrition provision at different life stages of mosquitoes on their life history, immunity and viral infection, whereas these three aspects and their results lack novelty. The authors also provided studies about their interactions which are interesting and have some novelty, like “does low nutrition in the larval stage can be rescued in the later adult stage?” but the manuscript doesn’t use enough words to emphasize this part. At least, from the Abstract people couldn’t grab the significance of this study and its novelty. Besides, the language of this manuscript should be improved. Some sentences are very confusing. The authors should be honest/careful when they claim “first” about their study (line 220, 275), as there are many nutrition studies about mosquitoes in different aspects and the authors also recognize this and have cited a lot of them. The result part is very tedious and difficult for people to read and understand, especially the immune gene expression part, adding one or two sentences about their main result will be much better. The discussion on the other hand, contains a lot of results but lacks information about the significance and implication of these results. For example, how does nutrition provision affect mosquito distribution and viral transmissibility? Besides, the methods are unclear about how many mosquitoes and replicates were tested in the study, did the authors have separate replicates about viral titers? How did they do non-infectious blood feeding? Did they use mock media? In general, the authors should put more effort into improving this manuscript.

Response: Thank you very much for the comments and suggestion. We agree with reviewer that the messaging of our manuscript needs improvement, especially the issues identified by both Reviewer #1 and #3 from methods, results and discussion. In light of these comments, we have intensively modified the abstract, result and discussion sections to clarify the issues.

Some small issues:
Line 39: how can adult nutritional stress affect adult body size?

Response: Larval and adult nutritional stress respectively affected adult body size and survival. The abstract has been rewritten for impact and clarity, and this particular sentence no longer occurs.

Line 133: what is the unit of female fecundity?
Response: The unit is count of eggs. We added this information now.

Reviewer #4 (Remarks to the Author):

The role of variation in relevant environmental factors on vector-borne disease transmission and the underlying mechanisms is an active field of investigation. Questions along this line are important as the majority of work done to date has focused on characterizing mosquito-pathogen interactions and mosquito immunity under highly controlled, laboratory conditions that are not reflective of variation mosquitoes may experience in the field. This study focuses on the effect of a biotic factor, such as food availability, in the larval and adult environment, and how variation in this factor affects mosquito fitness (survival and reproduction), immunity, and dengue vector competence. Strengths of the study include investigating the interactive effect of larval and adult nutrition, as well as infection status, on mosquito fitness and life history. Overall, the authors found that larval and adult nutrition and infection status had impact on these response variables. Weaknesses include
potential lack of biological replication, and how the authors executed the arbovirus infection and measured the immune response, which will require more in-depth discussion than what is currently presented. Thus, we have several major concerns that need to be addressed before this manuscript can be considered for publication in this journal.

Response: Thank you for reviewing our manuscript and recognizing the importance of our study. We have thoroughly modified the manuscript following your comments and suggestion.

Major Concerns:
• Justification for the sugar treatments being different- adults have access to sugar of differing concentrations, but access was ad libitum. How do we know that they consumed differing amount of sugar if compensatory feeding is occurring?

Response: Thank you for this good question. The possibility that mosquitoes with access to low sugar solution only may increase their feeding frequency/amount to compensate part of the energy deficit exists. Like many other studies, we offered the mosquitoes two cotton wicks soaked with different concentrations (in this case, 10% or 1%) sugar depending on the treatment and changed the cotton rolls daily. Such studies on the effects of sugar solution on mosquito life history traits or vector competence, with similar setting and even smaller concentration difference (as small as 2% and 4%), reported significant findings caused by the difference in sugar concentration (e.g., Lambrechts et al., 2006 Proc Royal Soc B 273, 1593; Vaidyanathan et al., 2008, Vector Borne Zoonotic Dis 8, 6; Lalubin et al., 2014, J Anim Ecol 83, 4; Sangare et al., 2014, Infect Genet Evol 25). Given our comparison is between 1% and 10% sucrose solution, and that we did notice effects of adult nutrition on a number of traits, we feel confident that if compensatory feeding occurred, this effect would have been limited. We did consider alternative methods of limiting access to sugar (e.g., access only during certain times), but felt that those had their own issues that would introduce greater variability. We do now raise this issue as a limitation in the discussion.

• Why only one bloodmeal? Especially since it is not clear how much aegypti rely on sugar sources in the field.

Response: This is another fair question. There are several elements to this decision, and we do realize a similar experiment could be performed that focused solely on differences in availability of blood feeding opportunities, or that at least made blood available on a frequent basis. One reason was logistics and the effort that would be involved in feeding females housed in individual cups with a Hemotek twice a week, which was beyond our capacity given that this was already a demanding experiment. We also felt it would introduce a potential loss of control, because individual variation in blood meal sizes could potentially compound. Perhaps most importantly, we find that feeding success on Hemoteks by *Ae. aegypti* is hit or miss – with the initial, single blood meal we can account for this by selecting blood fed females, but if they fail to take blood meals when offered later on for unclear reasons, that would introduce a loss of control. Although multiple bloodmeals and high dependency of blood rather than nectar as their energy source were reported from the field, these phenomena could be caused by interruption during their blood feeding due to host defense or environmental disturbance, and sugar-feeding in *Ae. aegypti* seems quite plastic depending on the availability of nectar and blood hosts. Here we did not seek to use multiple blood meals to replace/compensate sugar feeding and/or explore which combination of sugar dose and blood feeding frequency will

achieve the optimum fitness for the mosquitoes, but just use a full blood meal to enable mosquitoes to complete their life cycle and compare the effect of infectious and non-infectious blood meal on their fitness. We do address this now as a limitation in the discussion.

• We have substantial concerns about when immunity was measured with respect to infection and the lack of characterizing dynamics (of both the infection and the immune response). First, it was really unclear from the introduction and methods why gene expression 10 days after the challenge would be representative of the level of elicitation of immune signaling pathways during the infection process. Mosquito immune responses to infection are initiated 24-48 hr post-infection, depending on the pathogen, and involve midgut and systemic responses. It is unclear what characterizing the immune response at 10 days tells us about the response to infection. Further, characterizing infection phenotypes from mosquitoes that die naturally means we have a mixture of infection stages (midgut, disseminated, salivary glands) in the final sample leading to a lot of variation probably in the proportion of mosquitoes infected or with disseminated infections and the final titer. With this design,
there is a loss of mechanistic insight into where the bottle necks in infection are occurring and how the treatments affected the nature of these bottlenecks. These experimental design issues likely result in the disconnect between the effects of adult nutrition on dengue infection phenotypes with the effects of the larval environment on arbovirus immunity. In end, we still do not have a resolved picture of how the effects of these treatments connect across immunity and arbovirus susceptibility.

Response: We greatly appreciate the reviewer's concern. Ten days was selected as the time when disseminating infection could best be assessed in all treatment groups. Hence, the selection of 10d post infection was not done solely to describe dengue-derived elicited responses but rather to understand how the immune responsive profile varied in all treatment groups at this specific time, a stage when they are supporting disseminated viral infections. Furthermore, immune responses are not a single event and while the initial response (24-28 hr) are meant to suppress pathogens, we have less information on immune responses during chronic disseminated infections, especially when compounded by factors such as nutrition.
We have added a sentence to the methods section L541-542. The paragraph now reads:
"Ten days after blood feeding, these mosquitoes were frozen and stored at -80 °C for further processing. Ten days was selected as the time when disseminating infection could best be assessed in all treatment groups."

• The results section needs to rewritten to be explained in terms of the biological hypotheses instead of description of the model outputs. In particular, the interactions needs to be described. For example there is a three way interaction between LL, LA, and IB affecting fecundity (Line 139-140). What does that mean exactly? The effect of larval diet on fecundity depends on the nutrition the mosquito receives as an adult and whether the blood meal is infected or not...but how. Is it compounding? The results also are highly repetitive with the data that are presented in the figures and tables. The treatment means should appear in on or the other not both.

Response: Following both reviewer 1 and 4's suggestions, we have thoroughly modified the result section in order to fully interpret the biological implication of the data and reduce repeated description of data and model outputs. We also provide additional supplementary figures that help with visualization of the interactions.

• Replication? Not clear this was done rigorously. The only time replication is mentioned is in the immune assay section of the methods. For full biological replication to occur, the authors would have needed to replicate all experimental treatments (the larval portion of the experiment, the adult portion of the experiment, and the infections with different generations of mosquitoes). Thus, I suspect the replication they are referring to is actually pseudoreplication, which is a problem. Also, a clearer explanation of numbers of groups and sample sizes within each treatment and component of the study (life history, arbovirus infection, and immunity)

Response: The total sample sizes for each treatment are provided in Fig. 1. We have provided additional information on the experimental design in the methods, including that the experiment was performed in two subsequent blocks. Additionally, within each block, larvae were reared according to treatment level in 46 separate larval trays (each with 100 1st instars and kept under similar conditions), while adults were randomly assigned and initially housed and blood-fed in 20 different cages (L471-472). We also now provide detail on rotation of mosquito cages throughout the incubators to prevent any group effects associated with location within the incubator (L505-506). Respectfully, we think that since measurements of outcome variables were made on mosquitoes kept individually in small containers, that it is entirely reasonable to consider each individual as the experimental unit or biological replicate.

• The comparisons across treatments could also be clearer – especially in the adult stage, which consists of adults that receive high food but were from either a H / L food larval stage vs. adults that receive low food but were from either a H / L food larval stage. Sometimes adults are averaged across the larval stage…is this because the larval manipulation was not significant? If so, it would be helpful to state the non-significant effects as well as the significant effects. It would also be nice to know what your model structure is for each model (the factors included in the model were given, but you did not specify what interactions were explored or the models that were compared using AIC. In the supplement, it would be great to include this information. Also the summary tables are averaged across the main treatments and it would be nice to have all of the treatments broken out when presenting raw data means.

Response: we updated Table 1 and 2 to clearly present the suggested information about the different combinations of our three major treatments, that is, larval nutrition, adult nutrition and blood-feeding. We also added a Table S4 of models to provide the details of each model includes the global model and final model based on lowest AIC. All factors and interaction term in the model were specified.

• Finally, why not consider effects of the extrinsic incubation period? They open in the introduction by framing their study in terms of vectorial capacity. Vector competence has a fairly weak effect on overall transmission potential when compared to other variables in vectorial capacity, like EIP, when sensitivity analyses have been conducted. Variation in adult reserves and carry over effects on these reserves could also have big impacts on this response variable (e.g., see: https://parasitesandvectors.biomedcentral.com/articles/10.1186/s13071-014-0593-4 and https://royalsocietypublishing.org/doi/full/10.1098/rspb.2016.0298).

Response: We agree with the reviewer that extrinsic incubation period is an important factor in determining vectorial capacity and it would be interesting to explore the effect of larval and adult nutrition on EIP. Unfortunately, our study cannot measure this parameter from the ingestion of infectious blood meal to the time point that mosquitoes become infectious, because we must keep our mosquitoes alive until death to complete the survival assays. Due to its complexity nature, this study did not aim to measure all the factors that influence vectorial capacity, but just the impacts on life history traits, vector competence and immunity. However, we now mention EIP in the introduction (L94) and have highlighted this as an important area for future studies in the discussion (L447-451).

Minor Concerns:

• Introduction could be organized a bit differently. Focus second paragraph only on life history traits and third paragraph only on pathogen traits. Right now, the second paragraph has both. Also in the third paragraph, there is no description of EIP…which is significantly more important than vector competence.

Response: Thank you for the suggestion. We moved the sentences about pathogen traits to the new paragraph that identifies knowledge gaps between nutrition and vectorial capacity from the perspective of life history traits, infection (pathogen traits) and immunity. Although EIP is an important factor for vectorial capacity, we could not measure it in our study due to the need of survival assay. We note that we did describe the importance of the EIP in the context of the importance of survival throughout this period, and now make mention of the extrinsic incubation period explicitly in the introduction (L94) and the discussion (L447-451).

• Line 29- There has been previous work investigating the effect of larval nutrition on immunity in this species including study cited [29] and at least one looking at adult nutrition: https://journals.plos.org/plosntds/article?id=10.1371/journal.pntd.0005158. What we know from these studies and how this work differs needs to be more clearly spelled out in the introduction.

Response: Thank you for the recommendation. We referred these studies in the new paragraph of the introduction (L118-122) and compared our results with these studies in the discussion (L382-385).

• Line 63- First sentence is vague-

Response: we have rephrased this sentence.

• Line 89- if only a small proportion of mosquitoes are infected to we expect infection associated decreases in fecundity to really shape density?

Response: Here we stated that mosquito fecundity and survival could influence mosquito density (e.g., affected by diet), which is an important parameter in vectorial capacity equation. We cannot make the prediction that mosquito infection could shape mosquito density through its effect on fecundity, although this possibility exists, but we agree with the reviewer’s comment.

• Only one blood meal and associated egg lay were measured- how do they think this affects their survival and fecundity measures?

Response: This is a fair point, that we now address in a section on limitations of the current study. Whether the differences between treatments would change will require a different experimental design and we highlight the need for further study to address this.

• Figs 2, 5ab- again not clear what interactions really look like. Plotting data would be more helpful.

Response: We have added supplementary figures that show the interactions more clearly.

• Fig 3- it would be helpful to see all 8 treatments on a single plot or the 4 treatments broken up by larval nutrition.

Response: We have added a supplementary figure (Fig. S2) that follows the latter suggestion.

• Table 3- Not clear what this is a table of: are these the final model for each gene? So a "-" means that a parameter was not in the final model and the NS is that it was in the final model but not significant?

Response: Correct. We also explain this information in the table legend.

• Should cite: https://www.frontiersin.org/articles/10.3389/fmicb.2022.886787/full around line 234

Response: Cited.

• Does rpS7 change with experimental treatment? If so, this is not a good housekeeping gene (see https://royalsocietypublishing.org/doi/full/10.1098/rspb.2012.0638?rss=1). It also is unclear how the authors normalized expression of target genes to this gene?

Response: All the mosquitoes used in this study were reared and tested under the same standard temperature and humidity (see Methods). Therefore, the expression of S7 should be free of the effect of temperature. We normalized the expression of target gene to S7 by Livak and Schmittgen (2001)'s Delta Delta Ct method, using experimental mosquitoes' counterparts as the reference (see L548-558, Fig. 1, legends of Fig. 6 and Table 5).

Things to add to the discussion:

• Why does low adult food impact the probability of laying eggs only? I would expect low teneral

reserves to interact with low adult food to impact this response variable. There are multiple studies that have demonstrated that mosquitoes of low teneral reserves might need multiple bloodmeals to produce a batch of eggs (see https://pubmed.ncbi.nlm.nih.gov/22300806/). Also, how do you know all females were successfully mated?

Response: We kept male and female mosquitoes together for 3-5 days before conducting blood-feeding treatment and, like other studies, assume females were successfully mated. We added this sentence in method section (L480-481). We agree that the lack of effect of larval nutrition is surprising, but note that the effect of body size / larval nutrition on a pre-gravid state appears better established for *An. gambiae*. For *Ae. aegypti*, a paper by Scott et al cited in the above-mentioned review highlights geographic differences in the relationship between body size and multiple blood-feeding, which could suggest a genetic difference. We have added this topic to the discussion (L289-296).

• No effect of larval nutrition on vector competence…there has been a lot of work on this previously, showing that this should have an effect (Barry Alto's work). Why not here? Sugar provided ad libitum?

Response: We agree that a lot of studies have demonstrated a significant (or positive or negative) effect of larval nutrition on vector competence, including those done by Barry Alto's Lab. However, no effect of larval nutrition on vector competence was also reported, even including some work done by Barry Alto. For example, they found larval food quantity had no influence on the proportion of DENV-1-infected *Ae. aegypti*, and the titer of *Ae. aegypti* and *Ae. albopictus* infected mosquitoes (Buckner et al., 2016, J Med Ent 53:91-98). We cited these studies with both significant and non-significant effect and discussed why contradictory results were found, which is likely owing to the methodological difference. In our study however, we think that the effect of larval diet on vector competence might have operated indirectly through wing length (mosquito size), given that it was negatively associated with DENV infection rate. That is, larger-size mosquitoes reared at normal larval food, compared to those reared at low larval food, had lower DENV infection rate. We now describe this interpretation in more details in the discussion. Sugar was available continuously, but the interaction between larval and adult food was not significant here.

• Larval nutrition affects arbovirus immunity but adult nutrition affects DENV vector competence phenotypes…why? This mismatch and when the immune system was measured makes it hard to definitely say immunosuppression resulted in the reduced vector competence observed. Need to build

in justification for why 10 days is a relevant time point to measure and what this time point is capturing in your study (e.g., end point immunity)?

Response: Adult nutrition also affects arbovirus immunity in our study, although it is not as stark as larval nutrition. Given the complexity in the mechanism underlying vector competence, the 14 immune genes tested in our study may not cover all the factors that determine infection outcome. Therefore, a factor (e.g., larval nutrition) that has a strong impact on the expression of some immune genes may not necessarily show similar strong effects on vector competence.

Ten days was selected as the time when disseminating infection could best be assessed in all treatment groups. Hence, the selection of 10d post infection was not done solely to describe dengue-derived elicited responses but rather to understand how the immune responsive profile varied in all treatment groups at this specific time, a stage when they are supporting disseminated viral infections. Furthermore, immune responses are not a single event and while the initial response (24-28 hr) are meant to suppress pathogens, we have less information on immune responses during chronic disseminated infections, especially when compounded by factors such as nutrition.

• Need a limitations paragraph in the discussion section where you reflect on the limits of your study and implications for your results. Every study has limitations…these should be transparently addressed.

Response: We added a paragraph to discuss the limitations of our study (L274-282; L418-443).

Reviewers' comments:

Reviewer #1 (Remarks to the Author):

When I reviewed the original version of this manuscript I found it to be an experimentally impressive study with findings of significant importance. However, I found the manuscript to have messaging flaws and I offered several suggestions. The authors have revised the manuscript in a manner that addresses my comments. Below I expand.

1. On the messaging: the results section has been modified to go beyond the statistics and into the biology. Moreover, the discussion now discusses the results in relation to what is known. This manuscript now has a clearer—and important—message.

2. Seemingly contradictory statements: all the seemingly contradictory statements have been clarified. The clarification brought up one additional recommendation. The rebuttal letter clarifies that egg laying was measured in two ways: presence/absence (binary) and then the number of eggs laid by those that laid eggs (excluding non-egg layers). This approach makes sense. This is clearly spelled out in the rebuttal letter but not in the manuscript. Please clarify in the manuscript that the measurement of the number of eggs laid only considers those mosquitoes that laid eggs.

3. Tables: The updated tables are more informative than the original tables.

4. Stats: The statistical models are clearer.

5. Theories: The authors now consider these two theories (or evaluated their perceived relevance).

6. Firsts: The authors refrain from claiming firsts. Eliminating these claims does not detract from the significance if the study.

Importantly, the authors also now discuss the limitations of the study based on comments from the other reviewers. In defense of the authors, this is an already complex study where decisions had to be made. The other reviewers make salient (and correct) points regarding some of the decisions made and their limitations, but in my opinion, it would be logistically unfeasible for the authors to have created a study that addresses or considers all these limitations. The study, as it stands, is a significant advancement of our understanding of how larval and adult nutrition interact to shape mosquito fitness.

Some minor comments:

1. In the abstract, JAK-STAT is misspelled.

2. In lines 101-103, the definition of the mosquito immune system is overly simple. The immune system is more than just four signal transduction cascades. It also involves hemocytes, barrier defenses, constitutive defenses, etc.

3. In figure 1, it is unclear what the “brain heart infusion” in the egg stage signifies.

Reviewer #2 (Remarks to the Author):

The authors have properly addressed my queries. I have no further comment on what has been presented in this current version.

Reviewer #3 (Remarks to the Author):

The authors have satisfactorily responded to all my concerns

Reviewer #4 (Remarks to the Author):

Overall the manuscript is much improved. Thank you to the authors for their efforts on this front. However, I still have substantial concerns regarding the statistical analysis (not incorporating the hierarchical nature of the experimental design - see below) and the need for further clarification in the methods (experimental design, etc.) prior to this paper being accepted for publication.

Major comments

Clarifying methods- there are aspects of the methods which need to be clarified in order to make the experiment repeatable and allow the reader to assess the conclusions:

There is discordance between the text and Figure 1: The text on lines 471-482 indicates that there were a total of 92 larval trays each with 100 larvae. These 92 trays were split into two levels of nutrition (which would give 46 trays in each nutrition level and 4,600 larvae starting in each larval nutrition treatment). Individuals emerging from these trays (n is not mentioned in text) were split into adult nutritional treatments and then again into infection treatments. This does not match any of the n or n* supplied in figure Figure 1. Further it is not specified what n* denotes? It is also unclear what the Brain heart infusion is in this figure. Were larvae provided with a different diet prior to the laval treatments being imposed? Please update all sample sizes in text and figure to make it clear what the sample size is and what it refers to.

As it is written starting on line 508, females are individually tracked until death. Mosquitoes are checked for mortality twice daily and when discovered stored at -80 C. The wings were (later?) dissected on dry ice. It then appears that these dead mosquitoes are used to assay for infection and dissemination outcome using RT-qPCR. Is this accurate? At the moment it isn’t entirely clear that this was the case.

Methodological concerns:

There is not a citation for using mosquitoes that have been dead for up to 8 hours to detect and measure titer of dengue infection. This also means that mosquitoes are assayed for infection status and titer at varying time points depending on their survival. These infections are very dynamic over time and RNA degrades rapidly. Thus, I suspect that there could be a lot of variation in prevalence and titer depending on the amount of time a mosquito has been dead and when it was collected. It is not clear that including survival time is sufficient to control for these effects. How reliable is this technique? Alternatively, can more details justify and provide confidence that this is a good measure? This could dramatically affect the interpretation of their results and is not typically how infection prevalence and titer are measured in this field.

The argument of using individual mosquitoes because they are housed independently is incorrect. The experimental design is hierarchical, with experimental treatments occurring at different levels and nested within each other. If I understand the experimental design correctly, the experimental unit for the larval food manipulation is the tray (larvae within each tray are not independent from each other), for the DENV infection is the cage (adult mosquitoes within each cage are not independent from each other), and for the adult nutrition treatment the individual. The statistical analyses need to reflect the nested nature of these treatments or the authors are vastly inflating their power to detect significant differences through pseudoreplication. For this paper to go forward, I am strongly recommending a mixed effects model framework for each response variable measured that builds in random effects

that correspond with each level of treatment (Tray, Cage, and Individual mosquito). This could change the results, especially the ability to detect significance for some of the higher order interactions.

Replication - was not clear from the experimental design figure or the text that this was done in two experimental blocks. Clarify at multiple points throughout the ms please.

It is still unclear to me how relevant the sugar feeding treatment is for a highly anthropophilic vector. Much of the work done to date has found aegypti feeding primarily on blood and from humans. Please build in some justification to the introduction or the discussion on this matter. Varying the quality of the blood meal or providing multiple blood meals could yield a very different result.

Minor Comments:

The introduction was improved. On lines 111-122 it would be useful to discuss exactly what these found. For example, what did Telang et al. and Caragata determine about the effect of diet on expression?

https://journals.plos.org/plospathogens/article?id=10.1371/journal.ppat.1009870
Also still not citing everything in the area….lit review?

The three-way interactions are unclear. They need to be either unpacked in the appropriate part of the text.

The text on lines 238-244 is intriguing, but this should be in the discussion rather than the results.

Reviewers' comments:

Reviewer #1 (Remarks to the Author):

When I reviewed the original version of this manuscript I found it to be an experimentally impressive study with findings of significant importance. However, I found the manuscript to have messaging flaws and I offered several suggestions. The authors have revised the manuscript in a manner that addresses my comments. Below I expand.

Response: Thank you so much for the appreciation and constructive comments.

1. On the messaging: the results section has been modified to go beyond the statistics and into the biology. Moreover, the discussion now discusses the results in relation to what is known. This manuscript now has a clearer—and important—message.

Response: Agree.

2. Seemingly contradictory statements: all the seemingly contradictory statements have been clarified. The clarification brought up one additional recommendation. The rebuttal letter clarifies that egg laying was measured in two ways: presence/absence (binary) and then the number of eggs laid by those that laid eggs (excluding non-egg layers). This approach makes sense. This is clearly spelled out in the rebuttal letter but not in the manuscript. Please clarify in the manuscript that the measurement of the number of eggs laid only considers those mosquitoes that laid eggs.

Response: Thank you for the suggestion. We clarify this approach in the section of statistical analyses (L596-598) now.

3. Tables: The updated tables are more informative than the original tables.
Response: Agree.

4. Stats: The statistical models are clearer.
Response: Agree.

5. Theories: The authors now consider these two theories (or evaluated their perceived relevance).
Response: Thank you for your suggestion of testing these two theories.

6. Firsts: The authors refrain from claiming firsts. Eliminating these claims does not detract from the significance if the study.
Response: Agree.

Importantly, the authors also now discuss the limitations of the study based on comments from the other reviewers. In defense of the authors, this is an already complex study where decisions had to be made. The other reviewers make salient (and correct) points regarding some of the decisions made and their limitations, but in my opinion, it would be logistically unfeasible for the authors to have created a study that addresses or considers all these limitations. The study, as it stands, is a significant advancement of our understanding of

how larval and adult nutrition interact to shape mosquito fitness.
Response: Thank you for the comprehensive understanding of the strength and limitations of our study. We totally agree with the reviewer that some limitations need to be addressed in a different design of study in the future.

Some minor comments:

1. In the abstract, JAK-STAT is misspelled.
Response: corrected.

2. In lines 101-103, the definition of the mosquito immune system is overly simple. The immune system is more than just four signal transduction cascades. It also involves hemocytes, barrier defenses, constitutive defenses, etc.
Response: we added this information (L101-102) now.

3. In figure 1, it is unclear what the "brain heart infusion" in the egg stage signifies.
Response: it is the powder used for egg hatching. We added this in the figure legend now.

Reviewer #2 (Remarks to the Author):

The authors have properly addressed my queries. I have no further comment on what has been presented in this current version.

Response: Thank you very much for your time and comments.

Reviewer #3 (Remarks to the Author):

The authors have satisfactorily responded to all my concerns

Response: We really appreciate your inputs.

Reviewer #4 (Remarks to the Author):

Overall the manuscript is much improved. Thank you to the authors for their efforts on this front. However, I still have substantial concerns regarding the statistical analysis (not incorporating the hierarchical nature of the experimental design - see below) and the need for further clarification in the methods (experimental design, etc.) prior to this paper being accepted for publication.
Response: The improvement of the manuscript would not have been possible without your comments. We really appreciate that. We further modified our manuscript and responded to your concerns regarding the above issues as shown below.

Major comments

Clarifying methods- there are aspects of the methods which need to be clarified in order to make the experiment repeatable and allow the reader to assess the conclusions:

There is discordance between the text and Figure 1: The text on lines 471-482 indicates that there were a total of 92 larval trays each with 100 larvae. These 92 trays were split into two levels of nutrition (which would give 46 trays in each nutrition level and 4,600 larvae starting in each larval nutrition treatment). Individuals emerging from these trays (n is not mentioned in text) were split into adult nutritional treatments and then again into infection treatments. This does not match any of the n or n* supplied in figure Figure 1. Further it is not specified what n* denotes? It is also unclear what the Brain heart infusion is in this figure. Were larvae provided with a different diet prior to the laval treatments being imposed? Please update all sample sizes in text and figure to make it clear what the sample size is and what it refers to.

Response: Thank you for this question – we can see why this was confusing. The black n values indicated in Fig 1 refer to the pupae or adults that were used for the experiment, while the blue *n values refer separately to pupae and adults kept under similar conditions, but specifically for the gene expression study. We did indeed start off with a perhaps overly large set of larval trays. The reason for this was that we randomly selected pupae from among all the trays for each treatment and took only those forward in the experiment. Only individuals that pupated within a 7-9 day window after hatching were included to ensure specimens were of the same age. A further winnowing of individuals happened at the blood-feeding stage where only a subset of individuals fully engorged. To keep the balance between two block sessions, we randomly selected approximately 50 fully engorged individuals per treatment combination per block session so that each treatment combination and block session had the same sample size. Any treatment combination failed to get around 50 fully engorged individuals would fail the entire block session. To meet these experimental requirements, we prepared a much larger set of larvae to enable us to get the minimum number (~50) of experimental adults for each treatment combination. Since the black n values during larval and adult treatment stages may cause confusion, we have now removed them but only keep the final sample size for statistical analyses. We have added more details to the methods section and Fig. 1 clarifying this.

As it is written starting on line 508, females are individually tracked until death. Mosquitoes are checked for mortality twice daily and when discovered stored at -80 C. The wings were (later?) dissected on dry ice. It then appears that these dead mosquitoes are used to assay for infection and dissemination outcome using RT-qPCR. Is this accurate? At the moment it isn't entirely clear that this was the case.
Response: That's correct. We have modified the sentences (L526-527) to further clarify this information.

Methodological concerns:

There is not a citation for using mosquitoes that have been dead for up to 8 hours to detect and measure titer of dengue infection. This also means that mosquitoes are assayed for infection status and titer at varying time points depending on their survival. These infections are very dynamic over time and RNA degrades rapidly. Thus, I suspect that there could be a lot of variation in prevalence and titer depending on the amount of time a mosquito has been dead and when it was collected. It is not clear that including survival time is sufficient

to control for these effects. How reliable is this technique? Alternatively, can more details justify and provide confidence that this is a good measure? This could dramatically affect the interpretation of their results and is not typically how infection prevalence and titer are measured in this field.

Response: One of the main aims of our study was to figure out if feeding on DENV infectious blood meal shortens the life span of mosquitoes, while at the same time exploring how infection outcomes are impacted by time post-blood feeding under the influence of distinct levels of larval and adult nutrition. The typical way to measure infection outcomes, that is, sacrificing subsets of mosquitoes at certain time points post blood feeding to get the infection outcome and incomplete survival cannot meet the requirements of such study aim. Therefore, we adopted our current study design, which enabled us to get life-time infection outcome and survival data from the same mosquitoes. Maciel-de-Freitas et al. 2011 Trans. R. Soc. Trop. Med. Hyg. 105(8): 452-458 had used such design to assess the impact of DENV infection on mosquito survival. We cite this ref in our manuscript now (see L 527). Nonetheless, we agree with the reviewer that delayed detection of dead mosquitoes within the 8-hour observational interval could lower the observed titer in the follow-up DENV assay due to certain degree of RNA degradation, especially when the delayed detection events were not evenly distributed at each treatment combination. We have acknowledged this as an additional limitation of our study and call for a better approach to avoid delayed detection (e.g., hourly observation for dead mosquitoes, although it is labor intensive for observer and disturbing for resting mosquitoes) in the discussion section (L 448-456).

The argument of using individual mosquitoes because they are housed independently is incorrect. The experimental design is hierarchical, with experimental treatments occurring at different levels and nested within each other. If I understand the experimental design correctly, the experimental unit for the larval food manipulation is the tray (larvae within each tray are not independent from each other), for the DENV infection is the cage (adult mosquitoes within each cage are not independent from each other), and for the adult nutrition treatment the individual. The statistical analyses need to reflect the nested nature of these treatments or the authors are vastly inflating their power to detect significant differences through pseudoreplication. For this paper to go forward, I am strongly recommending a mixed effects model framework for each response variable measured that builds in random effects that correspond with each level of treatment (Tray, Cage, and Individual mosquito). This could change the results, especially the ability to detect significance for some of the higher order interactions.

Response: Thank you for the suggestion. Due to the following methodological and design considerations we respectfully disagree that tray and cage should be considered as the experimental unit for larval food manipulation and DENV infection, respectively.

As indicated above in the question regarding sample sizes, the experiment started off with a large number of larval trays that were kept under the same experimental conditions (except food between treatment levels), including trays, water, temperature, humidity, photocycle, larva density, etc.). Only a subset of pupae from each treatment level were selected (if they emerged within 7-9 days post-hatching), randomized in the same emergence cups, and allowed to emerge in the same cages (by larval treatment, and future adult nutritional treatment and dengue exposure). In other words, any potential tray effect is addressed through randomization.

For DENV infection, we randomly assigned half of the adults from each treatment level to either one of two cages for blood feeding, which only lasted for 45 min. Right after blood feeding, fully engorged mosquitos were randomly assigned to the same induvial cups for survival assay until death. Therefore, we believe any potential effects from the tray and cage might have been randomized or too transient to establish. We consider each individual mosquito as an independent biological replicate not only because they are individually caged during the measurement for life history traits, infection outcome and gene expression, but also because all the parameters were measured and compared at individual level, e.g., size, survival, fecundity, infection status and titer, gene expression level.

However, we did conduct our experiments for life history traits and infection outcome in two subsequent blocks (see the last sentence of each paragraph of the Methods and the legend of Fig. 1). We added block (I & II) as a fixed effect into our models for life history traits and infection outcome (L611-613). Although there were no qualitative change except the model for hazard ratios(Fig 3D), we updated all these results in this version of manuscript for its clarity.

Replication - was not clear from the experimental design figure or the text that this was done in two experimental blocks. Clarify at multiple points throughout the ms please.
Response: we add this information in the last sentence of each paragraph of the Methods and the legend of Fig. 1.

It is still unclear to me how relevant the sugar feeding treatment is for a highly anthropophilic vector. Much of the work done to date has found aegypti feeding primarily on blood and from humans. Please build in some justification to the introduction or the discussion on this matter. Varying the quality of the blood meal or providing multiple blood meals could yield a very different result.
Response: Although some studies reported *Ae. aegypti* feed primarily on blood and from human, there are also a number of field investigations revealed that this mosquito species can be easily attracted to and caught by sugar baits. Feeding on sugar or blood as their energy source might be highly flexible and depends on the habitats especially the relative availability of blood and sugar (see the last paragraph of discussion section). No doubt that multiple blood feeding is an interesting factor to explore, but adding this part of experiment was technically unfeasible for our study. We have acknowledged the limitations of not adding multiple blood feeding as an additional factor in the new paragraph of discussion section (L429-440). However, adding multiple blood feeding to our existing models may miss some controls caused by extra blood feeding like we stated in our last response and include extra assumptions for statistical analyses that would potentially weaken other parts of the study. Like Reviewer #1 stated, it would be logistically unfeasible for us to have created a study that addresses or considers all these limitations. We hope we can address this question in another study that requires a different design.

Minor Comments:

The introduction was improved. On lines 111-122 it would be useful to discuss exactly what these found. For example, what did Telang et al. and Caragata determine about the effect of diet on expression?

Response: We briefly mentioned what they have done and found in this research area, which leads to the knowledge gaps that we are studying in the introduction (L120-121) and the detailed discussion about what they found can be seen in the discussion section.

https://journals.plos.org/plospathogens/article?id=10.1371/journal.ppat.1009870
Also still not citing everything in the area….lit review?
Response: Thank you for the supplement. We already cited and discussed this important article in the main text (L 116-118, 348-352, 385-387).

The three-way interactions are unclear. They need to be either unpacked in the appropriate part of the text.
Response: The three-way interactions had been unpacked in Lines 159-163, 181-184, 276-280, 327-331 as well as Figure S1 and S2.

The text on lines 238-244 is intriguing, but this should be in the discussion rather than the results.
Response: Here we are reporting what we found and why, rather than interpreting its significance or comparing these findings with other studies. Moving this part to discussion will lead to a lack of full report of the results. Thus, we decide to keep them.

Reviewers' comments:

Reviewer #4 (Remarks to the Author):

Many aspects of the article has been improved from my previous review. However, one aspect that has not been taken into consideration that I have commented on twice now is related to the experimental design. I still have concerns here that need to be accommodated before I can recommend publication. The authors set up a hierarchical experimental design with experimental treatments nested within treatments. However, because the authors randomized across within each level instead of blocking they are unable to account for any variation that might be introduced due to each experimental unit (the tray or the cage, depending on the treatment) relative to their experimental treatments. That being said, this may be ok depending on the number of trays and cages in which animals were being pulled from, but this information is not present in the manuscript...or at least easily determined. Another problem is the authors continue to insist that the individual mosquito is a biological replicate, which is just not the case based on their experimental design. At minimum, since they likely have information on this, the cage in which they administered the DENV or uninfected bloodmeal could serve as the experimental unit. By treating individual as the experimental unit the authors are pseudoreplicating. For a good paper on this please see Hurlburt 1984 Pseudoreplication and the Design of Ecological Field Experiments. Pseudoreplication will inflate their power to detect differences or commit Type I errors, which is important here as they have several marginally significant results and higher order interactions. The degrees of freedom in their analysis need to be adjusted to reflect the cage as the experimental unit or to account for the hierarchy in their experimental design. Otherwise, I cannot recommend this for publication.

Reviewers' comments:
Reviewer #4 (Remarks to the Author):

Many aspects of the article has been improved from my previous review. However, one aspect that has not been taken into consideration that I have commented on twice now is related to the experimental design. I still have concerns here that need to be accommodated before I can recommend publication. The authors set up a hierarchical experimental design with experimental treatments nested within treatments. However, because the authors randomized across within each level instead of blocking they are unable to account for any variation that might be introduced due to each experimental unit (the tray or the cage, depending on the treatment) relative to their experimental treatments. That being said, this may be ok depending on the number of trays and cages in which animals were being pulled from, but this information is not present in the manuscript...or at least easily determined. Another problem is the authors continue to insist that the individual mosquito is a biological replicate, which is just not the case based on their experimental design. At minimum, since they likely have information on this, the cage in which they administered the DENV or uninfected bloodmeal could serve as the experimental unit. By treating individual as the experimental unit the authors are pseudoreplicating. For a good paper on this please see Hurlburt 1984 Pseudoreplication and the Design of Ecological Field Experiments. Pseudoreplication will inflate their power to detect differences or commit Type I errors, which is important here as they have several marginally significant results and higher order interactions. The degrees of freedom in their analysis need to be adjusted to reflect the cage as the experimental unit or to account for the hierarchy in their experimental design. Otherwise, I cannot recommend this for publication.

Response: Thank you very much for dedicating additional time to review our manuscript and for your thorough explanation of the issues. Ideally, we would prefer to have only one larva rearing tray and one cage per treatment level for rearing larvae and keeping mosquitoes during sugar and blood feeding. However, due to the necessity of obtaining a sufficient number of samples, this is nearly impossible. The concern here is that variations among trays or cages themselves, rather than the desired treatment effects, might impact the results, potentially leading to pseudo-replication issues if these undesired effects are significant.

To address this problem, we agree with the reviewer's suggestion of recording the ID of each tray and cage used to keep larvae and adults and treating them as experimental units or blocks in subsequent modeling. This would result in the number of samples for each treatment level being split into several subsections based on the number of experimental units or blocks. However, this approach might weaken the explanatory power of subsequent modeling.

To avoid this issue, we opted for a different approach: eradicating potential factors that could cause variations among trays and cages through standardization and randomization. In our study, all trays and cages were identical, with the same larval or adult density, food (sugar or blood per treatment level), rearing temperature, humidity, and photocycle (as described in the first subsection of the Methods). Additionally, randomization of individuals from different trays and cages was done before entering the next treatment or operation.

To avoid confusion caused by the winnowing process of mosquitoes from the start to the end of the experiment, we only presented the final sample size for each treatment level after blood feeding, as explained in previous rebuttal letter and shown in Figure 1. As a result, we did not include the numbers of trays and cages used for each treatment level in the main text. Nevertheless, this omission does not affect the understanding of the standardization and randomization process.

We firmly believe that potential factors that may cause variations among trays and cages have been effectively standardized or randomized. Although the possibility of pseudo-replication is low, we acknowledge this concern in the discussion (L447-454) and have added additional details to clarify our standardization and randomization measures for blood feeding in the method section (L520-521; 526-530).

The reviewer correctly recognized that standardization and randomization during larva and adult rearing can account for potential variations among trays and cages. We agree that the same standardization and randomization approach for blood feeding should be effective, especially considering that mosquitoes were only kept in the cages for 45 minutes.

Reviewers' comments:

Reviewer #4 (Remarks to the Author):

I agree that randomization will help take care of variation across trays and cages to some extent. However, the last experimental treatment was applied at the cage level and not the individual. So, at a minimum, the cage is the experimental unit here. By treating individuals as the experimental unit, the authors are pseudoreplicating (so the statement in the discussion that the chance that pseudoreplication could occur is low is completely inaccurate). By doing this, the analyses will have too many degrees of freedom, which is inflating the authors' ability to detect significant differences. This has implications for their results and conclusions, as there are many marginally significant effects and higher order interactions that could become non-significant once the analyses are adjusted. All I am asking is for the authors to rerun the analyses. It worries me that they will not consider this, especially since this is the more conservative approach. Unfortunately, I cannot recommend this paper for publication until this has been done.

Reviewers' comments:
Reviewer #4 (Remarks to the Author):

Many aspects of the article has been improved from my previous review. However, one aspect that has not been taken into consideration that I have commented on twice now is related to the experimental design. I still have concerns here that need to be accommodated before I can recommend publication. The authors set up a hierarchical experimental design with experimental treatments nested within treatments. However, because the authors randomized across within each level instead of blocking they are unable to account for any variation that might be introduced due to each experimental unit (the tray or the cage, depending on the treatment) relative to their experimental treatments. That being said, this may be ok depending on the number of trays and cages in which animals were being pulled from, but this information is not present in the manuscript...or at least easily determined. Another problem is the authors continue to insist that the individual mosquito is a biological replicate, which is just not the case based on their experimental design. At minimum, since they likely have information on this, the cage in which they administered the DENV or uninfected bloodmeal could serve as the experimental unit. By treating individual as the experimental unit the authors are pseudoreplicating. For a good paper on this please see Hurlburt 1984 Pseudoreplication and the Design of Ecological Field Experiments. Pseudoreplication will inflate their power to detect differences or commit Type I errors, which is important here as they have several marginally significant results and higher order interactions. The degrees of freedom in their analysis need to be adjusted to reflect the cage as the experimental unit or to account for the hierarchy in their experimental design. Otherwise, I cannot recommend this for publication.

Response: Thank you very much for dedicating additional time to review our manuscript and for your thorough explanation of the issues. Ideally, we would prefer to have only one larva rearing tray and one cage per treatment level for rearing larvae and keeping mosquitoes during sugar and blood feeding. However, due to the necessity of obtaining a sufficient number of samples, this is nearly impossible. The concern here is that variations among trays or cages themselves, rather than the desired treatment effects, might impact the results, potentially leading to pseudo-replication issues if these undesired effects are significant.

To address this problem, we agree with the reviewer's suggestion of recording the ID of each tray and cage used to keep larvae and adults and treating them as experimental units or blocks in subsequent modeling. This would result in the number of samples for each treatment level being split into several subsections based on the number of experimental units or blocks. However, this approach might weaken the explanatory power of subsequent modeling.

To avoid this issue, we opted for a different approach: eradicating potential factors that could cause variations among trays and cages through standardization and randomization. In our study, all trays and cages were identical, with the same larval or adult density, food (sugar or blood per treatment level), rearing temperature, humidity, and photocycle (as described in the first subsection of the Methods). Additionally, randomization of individuals from different trays and cages was done before entering the next treatment or operation.

To avoid confusion caused by the winnowing process of mosquitoes from the start to the end of the experiment, we only presented the final sample size for each treatment level after blood feeding, as explained in previous rebuttal letter and shown in Figure 1. As a result, we did not include the numbers of trays and cages used for each treatment level in the main text. Nevertheless, this omission does not affect the understanding of the standardization and randomization process.

We firmly believe that potential factors that may cause variations among trays and cages have been effectively standardized or randomized. Although the possibility of pseudo-replication is low, we acknowledge this concern in the discussion (L447-454) and have added additional details to clarify our standardization and randomization measures for blood feeding in the method section (L520-521; 526-530).

The reviewer correctly recognized that standardization and randomization during larva and adult rearing can account for potential variations among trays and cages. We agree that the same standardization and randomization approach for blood feeding should be effective, especially considering that mosquitoes were only kept in the cages for 45 minutes.

Response to the final change

- Please ensure that all Figures are cited in numerical order. Currently Figure 4 is cited before Figure 3 in the article file.

Response: Thank you for the careful reading. We now remove the citation of Fig. 4 at Line 153 and confirm that Fig. 3 is cited before Fig. 4.

Response to final changes

- The Reporting Summary document (which will be published) still looks rather strange. If possible, please
download a new form to fill out here: [https://www.nature.com/documents/nr-reporting-summary.pdf.](https://www.nature.com/documents/nr-reporting-summary.pdf)
Please note that this form is a dynamic 'smart pdf' and must therefore be downloaded and opened from a pdf reader such as Adobe Reader. This file will not open in an internet browser or by clicking the file.
Response: This link is not accessible by browser or Adobe Reader. We conducted a manual search for "reporting summary Communications Biology," downloaded the PDF using the Firefox browser, completed it using Adobe Reader, and uploaded it again. If you encounter any further issues, please provide specific details about the issues and send us the PDF via email. Thank you.

- Please upload the BioRender publication license documents for the illustrations in Figure 1.
Response: uploaded.

- Please add the name of the country to both author affiliations in the article file.
Response: done

- Please ensure that the figure titles do not reference specific panels. Figure panels should be mentioned in the legend only. Please correct this in the title for Figure 4 in the article file.
Response: done.

- Please ensure that all Figures are cited in numerical order. Currently Figure 4 is cited before Figure 3 in the article file.
Response: We conducted a thorough review of the article file and can confirm that Figure 3 is initially presented at line 167, with Figure 4 making its first appearance at line 192.

- Please cite Figures 6c, 6g-h, and 6l in the article file, or cite Figure 6 as a whole without citing the figure panels.
Response: Fig. 6I was mentioned in Line 237 and 249. We now add a sentence "There was no significant influence observed from any factors or interactions on the immune genes of Rel 1, PIAS, and Caspar (Fig. 6c, g & h; Table 3)." as the ending of the Results section.

All the above changes have been marked in red within the updated article file.
